# Expanding the molecular landscape of childhood apraxia of speech: evidence from a single-center experience

**DOI:** 10.3389/fnins.2024.1396240

**Published:** 2024-09-24

**Authors:** Daniela Formicola, Irina Podda, Elia Dirupo, Elena Andreucci, Sabrina Giglio, Paola Cipriani, Clara Bombonato, Filippo Maria Santorelli, Anna Chilosi

**Affiliations:** ^1^Department of Neurobiology and Molecular Medicine, IRCCS Fondazione Stella Maris, Pisa, Italy; ^2^Parole al Centro Studio di Logopedia, Genoa, Italy; ^3^Medical Genetics Unit, Meyer Children’s University Hospital IRCCS, Florence, Italy; ^4^Medical Genetics Unit, Department of Medical Sciences and Public Health, University of Cagliari, Cagliari, Italy; ^5^Department of Developmental Neuroscience, IRCCS Fondazione Stella Maris Scientific Institute, Pisa, Italy

**Keywords:** childhood apraxia of speech, exome sequencing, high confidence genes, low confidence genes, gene ontology and expression profile of CAS genes

## Abstract

**Background:**

Childhood apraxia of speech (CAS) is a genetically heterogeneous pediatric motor speech disorder. The advent of whole exome sequencing (WES) and whole genome sequencing techniques has led to increased identification of pathogenic variants in CAS genes. In an as yet uncharacterized Italian cohort, we aimed both to identify new pathogenic gene variants associated with CAS, and to confirm the disease-related role of genes already reported by others. We also set out to refine the clinical and neurodevelopmental characterization of affected children, with the aim of identifying specific, gene-related phenotypes.

**Methods:**

In a single-center study aiming to explore the genetic etiology of CAS in a cohort of 69 Italian children, WES was performed in the families of the 34 children found to have no copy number variants. Each of these families had only one child affected by CAS.

**Results:**

High-confidence (HC) gene variants were identified in 7/34 probands, in two of whom they affected *KAT6A* and *CREBBP*, thus confirming the involvement of these genes in speech impairment. The other probands carried variants in low-confidence (LC) genes, and 20 of these variants occurred in genes not previously reported as associated with CAS. *UBA6, ZFHX4*, and *KAT6A* genes were found to be more enriched in the CAS cohort compared to control individuals. Our results also showed that most HC genes are involved in epigenetic mechanisms and are expressed in brain regions linked to language acquisition processes.

**Conclusion:**

Our findings confirm a relatively high diagnostic yield in Italian patients.

## 1 Introduction

Childhood Apraxia of Speech (CAS) is a pediatric neurological motor speech disorder whose core features are lengthened and disrupted coarticulatory transitions, inconsistent errors on consonants and vowels, and abnormal word and phrase prosody due to impaired planning and programming of speech movements (American Speech-Language-Hearing Association, 2007). In CAS the precision and consistency of movements underlying speech are impaired in the absence of central or peripheral neuromuscular deficits and of structural oro-facial abnormalities. Clinically, CAS is diagnosed based on a qualitative assessment of speech samples gathered across different tasks, at the syllable, word, phrase and sentence level (see Supplementary Figure 1 for the operationalization of CAS signs). A set of speech features must be detected in the speech samples, such as difficulties in achieving the initial articulatory condition and in transitioning between different speech movements, increasing difficulties in multisyllabic sequences, syllable segregation, erroneous stress assignment, inconsistent devoicing, inconsistent errors with consonants and vowels, slow speech and diadochokinetic rate, groping, vocalic epenthesis and altered nasal resonance (Shriberg et al., 2011; Chenausky et al., 2020). These features result in poor intelligibility and effortful speech production. Typically, speech disruptions in CAS persist over time and necessitate protracted and intensive therapy. Ultimately, CAS can affect children’s expressive skills, academic performance (Miller and Lewis, 2022; Lewis et al., 2023) and participation in age-appropriate activities, as well as self-esteem and psychological well-being (Lewis et al., 2021; Cassar et al., 2023).

Although CAS presents in isolation in a relatively small proportion of cases, more often it co-occurs with prevalently expressive language impairment (Murray et al., 2019; Chilosi et al., 2022; Lewis et al., 2023), and with other complex neurodevelopmental disorders, such as Intellectual Disability (ID), Autism Spectrum Disorder (ASD), Attention Deficit Hyperactivity Disorder (ADHD) and Developmental Coordination Disorder (DCD) (Shriberg et al., 2019a; Stein et al., 2020; Chilosi et al., 2022; Chenausky et al., 2023; Morgan et al., 2024). CAS was estimated to occur in 1 child per 1,000 in the general pediatric population (Shriberg et al., 1997) and in 2.4% in a clinical population of children with speech sound disorders (Shriberg et al., 2019b). The prevalence was estimated to be higher in males than in females, with 2–3:1 male/female ratio (Hall et al., 1993; Lewis et al., 2004).

The etiology of CAS is still largely unknown. Unlike acquired apraxia of speech in adults, CAS is not linked to specific brain lesions, even though cerebral abnormalities are occasionally found (Chilosi et al., 2022; Chenausky et al., 2023; Chilosi et al., 2015). Nonetheless, advanced brain imaging techniques reveal volumetric and connectivity alterations in cortical areas and in subcortical networks underlying speech production (Kadis et al., 2014; Fiori et al., 2016; Conti et al., 2020).

A recent paper by Morgan et al. (2024) highlights that *FOXP2* dysfunction remains relatively specific to speech disorder, as compared with other recently identified monogenic conditions associated with CAS (Morison et al., 2023). In addition to *FOXP2* variants, single variants and small indels in other genes (such as *FOXP1* and *CNTNAP2*) and pathogenic copy number variants (CNVs) (e.g., 16p11.2 deletion) have been described in Chilosi et al. (2022). These variants often occur *de novo* and may involve genes already associated with other neurodevelopmental disorders, possibly determining phenotypic overlapping, with important implications for case management. The phenotypic spectrum can, therefore, be expanded to include “milder presentations,” defined by primary speech disorder in children with average cognitive performance (Morgan et al., 2024).

The advent of whole exome sequencing (WES) and whole genome sequencing techniques has led to increased identification of monogenic variants in CAS-associated genes. The list of these genes is provided in Supplementary Table 1. Despite the genetic heterogeneity of CAS, many implicated proteins functionally converge on pathways involved in chromatin modification or transcriptional regulation, opening the door to precision diagnosis and therapies (Morgan et al., 2024).

In a single-center study, we conducted WES analysis and deep phenotyping of speech and language profiles in a cohort of children with isolated CAS and no co-occurring neurodevelopmental disorders, except for language impairment. Our aim was to identify new pathogenic gene variants associated with CAS and to confirm the disease-related role of genes already reported by others. We also set out to refine the clinical and neurodevelopmental characterization of the affected children in our sample.

## 2 Materials and methods

### 2.1 Population study

Sixty-nine children (53 boys and 16 girls) were diagnosed with idiopathic CAS during evaluation for speech disorders at the Neurolinguistic and Neuropsychology Unit of IRCCS Stella Maris, a tertiary care hospital for children with neurological, neurodevelopmental, and psychiatric disorders. The CAS diagnosis was based on the presence of the three American Speech-Language-Hearing Association core speech characteristics^1^ and of at least five of the ten speech signs included in the Mayo checklist (Shriberg et al., 2011).

All the children underwent a comprehensive clinical and instrumental assessment that included structural brain imaging at 1.5 T and the standardized phenotypic assessment protocol that we adopt in complex neuropsychological and neurodevelopmental disorders (Chilosi et al., 2023).

Children were eligible for inclusion in the study if they were aged ≥4 years at clinical evaluation, if Italian was the only or primary language spoken at home, and if they were able to complete a full neurological and speech and language assessment. After an extensive neuropsychiatric evaluation by a specialized team, using specific procedures and carefully applying the DSM-5 clinical criteria, any children diagnosed with concomitant neurodevelopmental disorders such as ID, ASD and ADHD were excluded from the study. Other exclusion criteria were orofacial structural abnormalities liable to affect speech, hearing impairment, and the presence of “dysarthria only” speech features (Iuzzini-Seigel et al., 2022).

### 2.2 Deep speech and language phenotyping

Deep speech and language phenotyping was conducted through face-to-face evaluations by speech-language pathologists experienced in the assessment of pediatric motor speech disorders.

Speech evaluation included the following measures: (a) consonantal phonetic repertoire; (b) percentage of inaccurate productions of the items on a list of 46 probe words characterized by increasingly complex motor speech and syllable patterns; (c) percentage of inconsistent errors on the number of incorrectly produced probe words; (d) percentage of words with omitted syllables in the same probe word task; and (e) diadochokinetic (DDK) rate, as the number of fast repetitions of a nonsense three-syllable sequence (i.e., /pataka/). Overall intelligibility was rated by two independent scorers, who reviewed videos of speech samples and provided, independently, an intelligibility score ranging from 0 (never intelligible) to 5 (fully intelligible).

Language skills were assessed through the administration of standardized tests of receptive and expressive vocabulary and grammar. The level of expressive grammar complexity was evaluated according to the Grid for the Analysis of Spontaneous Speech (Chilosi et al., 2019). To estimate the overall level of speech and language proficiency, two composite severity scores were calculated based, respectively, on five speech and four language measures, as previously reported (Chilosi et al., 2022). Each measure was assigned a score of 0 when normal or borderline, and 1 when deficient. The maximum severity score was, therefore, 5 for speech and 4 for language. The speech composite severity score took into account consonantal phonetic repertoire, word inaccuracy and inconsistency of errors, syllable omissions, and DDK rate.

### 2.3 Cognitive assessment

Depending on the child’s age, non-verbal intelligence quotient (IQ) was assessed using the Wechsler Preschool and Primary Scale of Intelligence 3rd Edition (WPPSI-III) or the Wechsler Intelligence Scale for Children 3rd Edition (WISC-III) or 4th Edition (WISC-IV). Children were enrolled if their non-verbal IQ was >70.

### 2.4 Molecular studies

Prior to this study, all 69 patients had been investigated for pathogenic CNVs as part of their routine clinical assessment. The investigation was based on chromosome microarray analysis (CMA) and it revealed CNVs in 26 children (see Supplementary Table 2). Five of these children presented chromosome 16p11.2 deletion syndrome. Clinical data were insufficient in nine “negative” cases. In the remaining 34 cases, who tested negative for CNVs on CMA, we performed WES on the family trio. We prioritized cases that were completely negative for CNVs for exome sequencing. In 22 families the index case was the only affected individual, whereas in 10 kindred one of the two parents presented a history of speech language disorder. In a further family the father and brother of the index case also had a history of language delay. In the last family the father did not consent to molecular investigations (Supplementary Figure 2).

Total genomic DNA was extracted from peripheral blood using the QIAamp Mini Kit (QIAGEN®, Hilden, Germany) and samples were anonymized by means of numeric codes. Construction of enzymatic fragmentation libraries was followed by end repair, A-tailing, adapter ligation, and library amplification. Libraries were hybridized to a commercially available WES capture array (SeqCap EZ Exome v3, Nimblegen, Roche, Basel, Switzerland) and sequenced with NextSeq500/550 (Illumina Inc., San Diego, CA). The reads were aligned with the human reference hg19 genome using the Burrows-Wheeler Aligner (10.1093/bioinformatics/btp324), and mapped and analyzed with the IGV software package (Thorvaldsdottir et al., 2013). Downstream alignment processing was performed using the Genome Analysis Toolkit Unified Genotyper Module (Menke et al., 2016), SAMtool (Li et al., 2009), and Picard toolkit.^2^ Variants were annotated using Annovar (Wang et al., 2010) to obtain information such as variant frequency in different populations, and the predictions of the variant effect were obtained using a variety of software (CADD, SIFT, Polyphen2, MutationTaster, MutationAssessor, FATHMM and FATHMM MKL). Quality control of sequencing showed that 96% of the reads were mapped to the reference genome (hg19), and 97% of the targeted regions were covered by ≥30X reads with an average depth of 100X.

Clinically relevant germline variants were filtered step by step to identify the potentially interesting candidates; we retained only the single nucleotide variants and indel gene variants with a minor allele frequency (MAF) < 1% in the gnomAD v.2.1 database. Non-coding variants were excluded. In addition, we removed variants with a read depth < 20 and an alternative allele frequency < 35% of the normal allele (proportion of variant reads). Trio analyses were performed on the remaining gene variants by assuming a *de novo*, autosomal recessive, and X-linked mode of inheritance. We evaluated genes panel in-silico using the SFARI database and the Human Phenotype Ontology (HPO) of speech apraxia: HP: 0011098 and HP:0001249 to consider potential inherited variants from parents in light of genetic phenomena such as incomplete penetrance and variable expressivity. All reported variants were also inspected with IGV v2.7.

We analyzed exome data for loss-of-function (LoF) variants and predicted damaging missense variants. In particular, we focused on nonsense, missense, frameshift, and splice-region variant types, including synonymous variants, with a MAF < 0.03% in the gnomAD v2.1 database, in consideration of potentially causal variants for CAS.

The genes were further screened to assess biological significance for neural development, and known neurological disorders and functions, using the Online Mendelian Inheritance in Man (OMIM) database and consulting the PubMed website.

To verify whether detected variants were located in genes already known to be associated with ASD or neurodevelopmental disorders, we scrutinized the SFARI database.^3^

Classification of variants complied with the American College of Medical Genetics (ACMG) guidelines (Richards et al., 2015), and variants were therefore split into two categories (Hildebrand et al., 2020) according to whether they occurred in high-confidence (HC) or low-confidence (LC) genes.

The HC variants met the following criteria: (i) LoF variants (nonsense, frameshift and splicing variant); for splicing variants we retained those with SpliceAI scores >0.2; for damaging missense variants we retained those satisfying at least one of the following conditions: CADD Phred score ≥ 20 or a Revel score ≥ 0.644; (ii) variant located in genes associated with CAS phenotype, and in which segregation analysis was supportive.

Variants were defined LC if they involved (i)OMIM genes not associated with disease, (ii) class 3 variants in the ACMG classification, (iii) or class 4 variants located in genes not fully consistent with the proband’s phenotype, (iiii) or when family segregation was not supportive.

#### 2.4.1 Gene ontology analysis

For the HC gene list in our analyses, we queried the TOPPGENE database to perform a Gene Ontology (GO) analysis, focusing on identifying enriched biological processes, cellular components, and molecular functions associated with the genes. We also conducted a comprehensive set enrichment analysis based on the Human Phenotype Ontology (HPO), cellular components, and biological processes. This approach allowed us to identify relevant biological pathways and cellular components associated with our gene set, following methodologies similar to those in the recent studies by Zhang et al. (2023) and Maleki et al. (2020).

#### 2.4.2 Gene expression data analysis

The Gene-level RNA-seq RPKM expression data were retrieved from the Developmental.

Transcriptome section^4^ of the BrainSpan Atlas of the Developing Human Brain database (Miller et al., 2014). Two custom selections were generated, for the HC and LC gene lists, respectively. Data were sorted by structure, and the resulting expression matrices were downloaded. The downloaded files were subsequently imported into R, and heatmaps were generated for visualization employing the ComplexHeatmap package.^5^

## 3 Results

**Figure 1:**
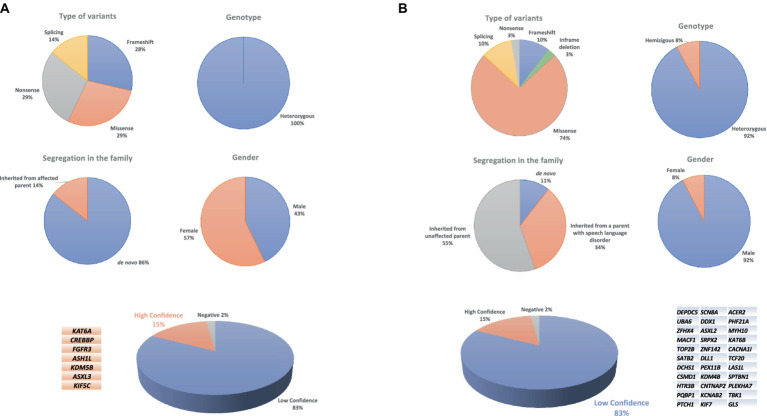
Chart of high-confidence **(A)** and low-confidence variants **(B)** in a CAS cohort.

The 34 probands who underwent WES analysis had a mean age of 71.8 ± 26.2 months (range: 47–159 months). WES studies did not identify any HC or LC variants in one child (proband #60). Instead, HC variants were identified in 7 children, 3 males and 4 females (Figure 1A). The mean age of this HC group was 67 ± 19.1 months (range: 50–94 months). The variants affected 7 different genes (Table 1). LC variants (Figure 1B; Table 2), on the other hand, were detected in 26 children, 24 males and 2 females, aged 73.1 ± 28.4 months (range: 47–159 months). Table 3 synthesizes the comparisons between the clinical characteristics of the children with HC and LC variants. Whilst speech severity did not differ significantly between the two groups, language impairment was more frequent and more severe in the HC than in the LC group. Moreover, in the HC group language impairment involved more frequently also receptive language skills. Tables 4, 5 report the phenotypic characteristics of the children belonging to the two groups.

**Table 1:** High-confidence gene variants in individuals with CAS.

Proband number	Sex	Chr:Pos (GRCh37)	Gene (transcript)	OMIM gene	Phenotype MIM number	SFARI	DNA variant	Zygosity	Protein change	Effect	*In silico* predictions	ACMG Classification	gnom ADExome v2.1 ~	Inheritance	Reference*
27	M	8:41798633	*KAT6A (NM_006766.5)*	608,570	#616268	2S	c.2765_2766insG	Htz	p.Ser923PhefsTer2	Frameshift	gnomADpLI = 1	Pathogenic (PVS1 very strong, PM2 supporting, PS2 supporting)	0	*De novo*	PMID: 28636259
28	M	16:3779691	*CREBBP (NM_004380.3)*	600,140	#618332 #180849	1	c.5357G > A	Htz	p.Arg1786His	Missense	Missense Z-score = 3.09, Revel = 0.71, CADD = 29.1	Likely pathogenic (PM2 supporting, PM1 moderate, PS2 supporting, PP2 supporting, PM5 supporting, PP3 supporting)	0	*De novo*	PMID: 27311832
32	F	4:1803571	*FGFR3 (NM_000142.5)*	134,934	#602849	NA	c.749C > G	Htz	p.Pro250Arg	Missense	Revel = 0.772 CADD =21.2	Likely pathogenic (PM2 supporting, PP3 supporting, PS3 supporting, PS2 strong)	0.00000418	*De novo*	PMID: 24168007; 26,740,388; 20,301,588
33	F	1:155448537	*ASH1L (NM_018489.3)*	607,999	#617796	1	c.4124del	Htz	p.Ser1375IlefsTer22	Frameshift	gnomADpLI = 1	Pathogenic (PVS1 very strong, PM2 supporting, PS2 supporting)	0	*De novo*	PMID: 34373061; 24,267,886, 25,363,768; 8,191,889; 28,394,464
34	F	1:202702681	*KDM5B (NM_006618.5)*	605,393	#618109	1	c.3757C > T	Htz	p.Arg1253Ter	Nonsense	CADD = 41	Pathogenic (PVS1 very strong, PM2 supporting, PS2 supporting)	0	*De novo*	PMID: 33350388
35	M	18:31325075	*ASXL3 (NM_030632.3)*	615,115	#615485	1	c.5263C > T	Htz	p.Gln1755Ter	Nonsense	gnomADpLI = 1, CADD = 40	Likely pathogenic (PVS1 strong, PM2 supporting, PS2 supporting)	0	*De novo*	PMID: 33392332
37	F	2:149866866	*KIF5C (NM_004522.3)*	604,593	#615485	2S	c.2767 + 1G > T	Htz	NA	Splicing donor site	gnomADpLI = 1 spliceAI DL = 1	Likely pathogenic (PVS1 very strong, PM2 supporting)	0	Inherited from affected mother	PMID: 23033978- PMID: 24812067

**Table 2:** Low-confidence variants in individuals with CAS.

Proband number	Sex	Chr:Pos (GRCh37)	Gene (transcript)	OMIM	Phenotype MIM number	SFARI	DNA variant	Zygosity	Protein change	Effect	*In silico* predictions	ACMG/AMP Classification	gnom ADExome v.2.1 ~	Inheritance
29	M	12:64879786	*TBK1 (NM_013254.4)*	617,900	#616439 #620880	NA	c.1330dup	Htz	p.Arg444ThrfsTer5	Frameshift	gnomADpLI = 0.99	Likely Pathogenic (PVS1 very strong, PM2 supporting)	0	Inherited from affected mother
30	M	8:2823296	*CSMD1 (NM_033225.6)*	608,397	–	2	c.9280 + 1G > A	Htz	NA	Splice Acceptor Site	gnomADpLI = 1 spliceAI DL = 1	No known gene-disease association	0	*De novo*
31	M	X:99917252	*SRPX2 (NM_014467.3)*	300,643	#300643	NA	c.296 G > T	hmz	p.Ser81Arg	Missense	Revel = 0.808 CADD = 28	Uncertaine SIgnificance (PM2 supporting, PP3 moderate, PP4 Moderate)	0	Inherited from unaffected mother
36	M	11:16838781	*PLEKHA7 (NM_001329630.2)*	612,686	–	NA	c.1432C > T	Htz	p.Arg478Ter	Nonsense	CADD = 34	No known gene-disease association	0.000016	*De novo*
38	F	22: 32218728	*DEPDC5 (NM_001242896.3)*	614,191	#604364 #620504	S	c.2056A > G	htz	p.Ser686Gly	Missense	Missense Z-score = 3.2858 Revel = 0.029 CADD = 17.03	Uncertain significance (PM2 supporting, BP4 supporting)	0	*De novo*
39	M	4: 68543354	*UBA6 (NM_018227.6)*	611,361	–	NA	c.440A > G	htz	p.Asp147Gly	Missense	Revel = 0.238 CADD =24.4	No known gene-disease association	0	Inherited from father with a history of speech language disorder
40	M	4:68484765	*UBA6 (NM_018227.6)*	611,361	–	NA	c.3109G > T	htz	p.Asp1037Tyr	Missense	Revel = 0.501 CADD = 31	No known gene-disease association	0	Inherited from unaffected mother
41	M	8:77616397	*ZFHX4 (NM_024721.5)*	606,940	#178300	NA	c.74C > G	htz	p.Thr25Arg	Missense	Revel = 0.161 CADD = 23.5	Uncertain significance (PM2 supporting)	0	Inherited from father with dyslexia
42	M	1: 3983824	*MACF1 (NM_012090.5)*	608,271	#618325	3S	c.7003_7005del	htz	p.Ala2335del	Inframe deletion	gnomADpLI = 1	Uncertain significance (PM2 supporting, PM4 moderate)	0	Inherited from father with fluency disorder
43	M	3: 25656718	*TOP2B (NM_001330700.2)*	609,296	#609296	S	c.3569C > T	htz	p.Ala1190Val	Missense	Missense Z = 3.86 Revel = 0.22 CADD = 24	Uncertain significance (PM2 supporting, PP2 supporting, PS2 supporting)	0,00003935	*De novo*
44	M	2: 200188563	*SATB2 (NM_015265.4)*	608,148	#612313	2	c.1505A > C	htz	p.Gln502Pro	Missense	Missense Z = 4.05 Revel =0.655 CADD = 26.9	Uncertain significance (PM2 supporting, PP2 supporting, PP3 supporting, PM1 supporting)	0	Inherited from mother with speech language disorder
		11: 6648209	*DCHS1 (NM_003737.4)*	603,057	#607829 #601390	NA	c.6061C > T	htz	p.Arg2021Cys	Missense	Revel = 0.434 CADD = 32	Uncertain significance (PM2 supporting)	0	Inherited from unaffected father
		8: 3072060	*CSMD1 (NM_033225.6)*	608,397	–	2	c.4826A > G	htz	p.Asp1609Gly	Missense	Revel = 0.533 CADD = 24.1	No known gene-disease association	0	Inherited from unaffected father
45	M	11: 6644979	*DCHS1 (NM_003737.4)*	603,057	#607829 #601390	NA	c.7928C > T	htz	p.Ser2643Leu	Missense	Revel =0.489 CADD = 26.9	Uncertain significance (PM2 supporting)	0	Not available
46	M	11:113802099	*HTR3B* (NM_006028)	604,654	–	NA	c.214-7C > T	htz	–	Splicing region	SpliceAI = 0.00	No known gene-disease association	0	Inherited from unaffected father
		X: 48759725	*PQBP1 (NM_001032382.2)*	300,463	#309500	NA	c.508C > T	hmz	p.Arg170Trp	Missense	Revel = 0.386 CADD = 24.6;	Uncertain significance (PM2 supporting, BS2 supporting)	0	Inherited from unaffected mother
47	M	11:113802099	*HTR3B* (NM_006028)	604,654	-	NA	c.214-7C > T	htz	-	Splicing region	SpliceAI = 0.00	No known gene-disease association	0	Inherited from unaffected father
		9: 98270641	*PTCH1(NM_000264.5)*	601,309	#610828 #109400	1	c.4del	htz	p.Ala2ProfsTer78	Frameshift	gnomADpLI = 1	Uncertain significance (PVS1 strong, PM2 supporting)	0	Inherited from unaffected mother
		12: 52162728	*SCN8A (NM_001330260.2)*	600,702	#614306 #614558 #617080	1	c.2981 T > A	htz	p.Met994Lys	Missense	Missense Z = 7.64 Revel = 0.8 CADD = 23.8	Uncertain significance (PM2 supporting, PP2 supporting, PP3 supporting, PM1 supporting)	0	Inherited from unaffected mother
48	M	2:15735661	*DDX1 (NM_004939)*	601,257	–	NA	c.116G > A	htz	p.Gly39Glu	Missense	Revel = 0.677 CADD = 29.2	No known gene-disease association	0	Inherited from unaffected father
		2: 25982429	*ASXL2 (NM_018263)*	612,991	#617190	NA	c.861C > G	htz	p.Ile287Met	Missense	Revel = 0.314 CADD = 23.5	Uncertain significance (PM2 supporting)	0,00000401	Inherited from unaffected father
49	M	2:219510928	*ZNF142 (NM_001379659.1)*	604,083	#618425	NA	c.2017C > T	htz	p.Arg673Cys	Missense	CADD = 24.8	Uncertain significance (PM2 supporting)	0,0000681	Inherited from unaffected father
50	M	6:170593000	*DLL1 (NM_005618.4)*	606,582	#618709	2S	c.1367G > A	htz	p.Gly456Asp	Missense	Revel = 0.825 CADD = 24.1	Uncertain significance (PM2 supporting, PP3 moderate)	0	Inherited from unaffected father
		1:145522804	*PEX11B (NM_003846.3)*	603,867	#614920	NA	c.665_672del	htz	p.L222PfsTer88	Frameshift	–	Uncertain significance (PVS1 strong, PM2 supporting)	0	Inherited from unaffected father
51	M	19: 5151508	*KDM4B (NM_015015.3)*	609,765	#619320	2	c.3277G > A	htz	p.Gly1093Arg	Missense	Missense Z = 3.48 Revel = 0.069 CADD:25.4	Uncertain significance (PM2 supporting, PP2 supporting, BP4 supporting)	0	Inherited from father with a history of speech language disorder; a brother is also affected
52	M	7:147600801	*CNTNAP2 (NM_014141.6)*	604,569	#610042	2S	c.2243A > G	htz	p.Asp748Gly	Missense	Revel = 0.356 CADD = 25.3;	Uncertain significance (PM2 supporting, BP1 supporting)	0	Inherited from father with a history of speech language disorder
		1: 6155616	*KCNAB2 (NM_001199862.2)*	601,142	–	NA	c.880C > A	htz	p.Leu294Met	Missense	Revel = 0.354 CADD = 26.0	No known gene-disease association	0	Inherited from unaffected mother
53	M	9:98248069	*PTCH1 (NM_000264.5)*	601,309	#610828 #109400	1	c.482C > G	htz	p.Thr161Ser	Missense	Revel = 0.693 CADD = 25.5	Uncertain significance (PM2 supporting, PP3 supporting)	0	Inherited from unaffected father
54	M	15: 90176938-GCTCATTTCTGC-G	*KIF7 (NM_198525.3)*	611,254	#607131 #614120 #200990	NA	c.2560_2570del	htz	p.Ala854GlnfsTer14	Frameshift	–	Likely pathogenic (PVS1 very strong, PM2 supporting)	0	Inherited from father with a history of speech language disorder
		9: 19423859	*ACER2 (NM_001010887.3)*	613,492	–	NA	c.109-1G > C	htz	–	Splicing region	SpliceAI acceptor loss = 0.99	No known gene-disease association	0	Inherited from father with a history of speech language disorder
55	F	11:45957270	*PHF21A (NM_001352027.3)*	618,725	#618725	1S	c.1705A > C	htz	p.Lys569Gln	Missense	CADD = 27.7	Uncertain significance (PM2 supporting)	0	Inherited from mother with a history of speech language disorder
		17: 8383527	*MYH10 (NM_001256012.3)*	160,776	–	2	c.5498A > G	htz	p.Gln1833Arg	Missense	Missense Z = 5.01 Revel = 0.413 CADD = 22.1	No known gene-disease association	0	inherited from mother with a history of speech language disorder
56	M	10:76789416	*KAT6B (NM_012330.4)*	603,736	#606170 #603736	3	c.4834C > T	htz	p.Arg1612Cys	Missense	Revel = 0.612 CADD = 27.5	Uncertain significance (PM2 supporting)	0	Inherited from unaffected father
		22: 40060756	*CACNA1I (ENST00000402142.3)*	608,230	#620114	2	c.3679G > A	htz	p.Gly1227Ser	Missense	Missense Z = 4.64 Revel = 0.9589 CADD = 28.7	Likely pathogenic PM2 supporting, PP2 supporting, PP3 strong	0	Inherited from unaffected father
57	M	22: 42606366	*TCF20 (NM_001378418.1)*	603,107	#618430	1S	c.4946 T > C	htz	p.Ile1649Thr	Missense	Revel = 0.600 CADD = 27.0	Uncertain significance (PM2 supporting)	0	Inherited from unaffected mother
58	M	X: 64734848	*LAS1L (NM_031206.7)*	300,964	#309585	3	c.1933G > A	hmz	p.Val645Met	Missense	Revel = 0.103 CADD = 24.8	Uncertain significance (PM2 supporting, BP4 supporting)	0	Inherited from unaffected mother
		2: 54876909	*SPTBN1 (NM_003128.3)*	182,790	#619475	2S	c.5360G > C	htz	p.Trp1787Ser	Missense	Revel = 0.762 CADD = 32	Uncertain significance (PM2 supporting, PP2 supporting, PP3 supporting, PM1 supporting)	0	Inherited from unaffected mother
59	M	2:191797566	*GLS (NM_001256310.2)*	138,280	#618339 #618328 #618412	NA	c.1775A > G	htz	p.Glu592Gly	Missense	Missense Z = 3.73 Revel = 0.234 CADD = 22.3	Uncertain significance (PM2 supporting, PP2 supporting, BP4 supporting)	0	Inherited from mother with a history of speech language disorder

**Table 3:** Comparison between clinical characteristics of children with HC and LC gene alterations.

	High confidence	Low confidence	Significance
Probands	7	26	
Male/female	3/4 (ratio 1:1.33)	24/2 (ratio 1:12)	***p* = 0.003**
Mean age	67, SD = 19.1 months (range: 50–94 months).	73.1, SD = 28.4 months (range: 47–159 months).	
Non-verbal IQ	95.4, SD = 11.9	106.3, SD = 17.4	*p* = 0.075
Speech composite severity score	4.7, SD = 0.4	4.5, SD = 0.5	χ^2^ = 0.41, *p* = 0.81
Language composite severity score	3.7, SD = 0.4	2.1, SD = 1.1	**χ**^**2**^ **= 12,2, *p* = 0.016**
Probands with co-occurrent Language Impairment	7	25	
Percentage of probands with receptive-expressive language impairment	100%	44%	**χ**^**2**^ **= 7.4, *p* = 0.025**

The significant association is in bold.

**Table 4:** Clinical features of individuals with CAS and high-confidence variants.

Proband Number	Sex	Chr:Pos (GRCh37)	Gene (transcript)	Disorder	Speech composite severity score*	Language composite severity score**	Performance intelligence quotient	Early feeding difficulties	Brain MRI	Other information
27	M	8:41798633	*KAT6A (NM_006766.5)*	CAS, receptive-expressive language impairment, normal non-verbal IQ	4	3	89	Yes	Asymmetry of lateral ventricles, C1—C2 synostosis but not syringomyelia	Severe convergent strabismus, bilateral foot syndactyly
28	M	16:3779691	*CREBBP (NM_004380.3)*	CAS, receptive-expressive language impairment, normal non-verbal IQ	4	3	111	No	Normal	Bifid incisors, positional clubfoot and metatarsus varus corrected with orthopedic shoes, mild ligament hyperlaxity, mild delay in static and dynamic balance and in fine hand movements on Movement-ABC2
32	F	4:1803571	*FGFR3 (NM_000142.5)*	CAS, receptive-expressive language impairment, normal non-verbal IQ	5	4	85	Yes	Normal	Allergy to milk, egg white, wheat, beans and tomatoes. Macrosomia (weight 95^th^ perc., height 90^th^ perc., head circumference 98^th^ perc.), mild oral apraxia.
33	F	1:155448537	*ASH1L (NM_018489.3)*	CAS, receptive-expressive language impairment, normal non-verbal IQ	5	4	89	No	Normal	Mild exophoria of the right eye
34	F	1:202702681	*KDM5B (NM_006618.5)*	CAS, receptive-expressive language impairment, normal non-verbal IQ	5	4	98	No	Normal	None
35	M	18:31325075	*ASXL3 (NM_030632.3)*	CAS, receptive-expressive language impairment, normal non-verbal IQ	5	4	107	No	Normal	None
37	F	2:149866866	*KIF5C (NM_004522.3)*	CAS, receptive-expressive language impairment, borderline non-verbal IQ	5	4	78	No	Normal	None

*Speech composite severity score: range 0 (no impairment) – 5 (severe impairment across all the speech measures). Each impaired speech measure (consonantal phonetic repertoire, inaccuracy, inconsistency, syllable omissions, diadochokinetic rate) contributes one point to the composite score.

**Language composite severity score: range 0 (no impairment) – 4 (severe impairment across all the language measures). Each impaired language measure (expressive and receptive lexicon, expressive and receptive grammar) contributes one point to the composite score.

**Table 5:** Clinical features of individuals with CAS and low-confidence variants.

Proband number	Sex	Chr:Pos (GRCh37)	Gene (transcript)	Disorder	Speech composite severity score *	Language composite severity score **	Performance intelligence quotient	Early feeding difficulties	Brain MRI	Other information
29	M	12:64879786	*TBK1 (NM_013254.4)*	CAS, receptive-expressive language impairment, borderline non-verbal IQ.	5	3	78	No	Normal	Developmental coordination disorder, macrosomia (weight 99^th^ percentile, height 95^th^ percentile, head circumference 98^th^ percentile). Lower limb dysmetria
30	M	8:2823296	*CSMD1 (NM_033225.6)*	CAS, expressive language impairment, normal non-verbal IQ	4	1	107	Yes	Normal	Mild limb hypotonia
31	M	X:99917252	*SRPX2 (NM_014467.3)*	CAS, receptive-expressive language impairment, normal non-verbal IQ	5	3	89	Yes	Normal	Macrocrania and prominent frontal bumps
36	M	11:16838781	*PLEKHA7 (NM_001329630.2)*	CAS, receptive-expressive language impairment, normal non-verbal IQ	3	3	91	No	Normal	None
38	F	22: 32218728	*DEPDC5* (NM_001242896.3)	CAS, expressive language impairment, normal non-verbal IQ	5	2	102	No	Normal	None
39	M	4: 68543354	*UBA6* (NM_018227.6)	CAS, expressive grammar impairment, normal non-verbal IQ	4	1	117	No	“low” cerebellar amygdalae (but no Chiari 1)	None
40	M	4:68484765	*UBA6* (NM_018227.6)	CAS, expressive language impairment, normal non-verbal IQ	4	3	104	No	Normal	Oral apraxia, mild ligament hyperlaxity
41	M	8:77616397	*ZFHX4* (NM_024721.5)	CAS, expressive grammar impairment, normal non-verbal IQ	4	1	139	No	“low” cerebellar amygdalae (but no Chiari 1)	Frequent middle ear infections and colds
42	M	1: 3983824	*MACF1* (NM_012090.5)	CAS, expressive grammar impairment, normal non-verbal IQ	4	1	123	Yes	Normal	None
43	M	3: 25656718	*TOP2B* (NM_001330700.2)	CAS, expressive grammar impairment, normal non-verbal IQ	5	1	127	No	Hydrocele	None
44	M	2: 200188563	*SATB2* (NM_015265.4)	CAS, receptive-expressive language impairment, borderline non-verbal IQ	5	3	81	No	Normal	Oral apraxia, developmental coordination disorder, pectus excavatum, poorly represented panniculus adiposus, elongated face, drooling
		11: 6648209	*DCHS1* (NM_003737.4)							
		8: 3072060	*CSMD1* (NM_033225.6)							
45	M	11: 6644979	*DCHS1* (NM_003737.4)	CAS, receptive-expressive language impairment, normal non-verbal IQ	5	3	104	Yes	Normal	Micrognathia, protruding ears, poorly represented panniculus adiposus
46	M	11:113802099	*HTR3B* (NM_006028)	CAS, receptive-expressive language impairment, normal non-verbal IQ	4	4	104	No	Normal	Mild hypotonia and ligament hyperlaxity
		X: 48759725	*PQBP1* (NM_001032382.2)							
47	M	11:113802099	*HTR3B* (NM_006028)	CAS, no language impairment, normal non-verbal IQ	4	0	137	No	Normal	None
		9: 98270641	*PTCH1* (NM_000264.5)							
		12: 52162728	*SCN8A* (NM_001330260.2)							
48	M	2:15735661	*DDX1* (NM_004939)	CAS, expressive grammar impairment, normal non-verbal IQ	5	1	122	No	Normal	None
		2: 25982429	*ASXL2* (NM_018263)							
49	M	2:219510928	*ZNF142* (NM_001379659.1)	CAS, expressive language impairment, normal non-verbal IQ				No	Normal	Celiac disease, preterm birth at 31 + 4 weeks
50	M	6:170593000	*DLL1* (NM_005618.4)	CAS, expressive grammar impairment, normal non-verbal IQ	4	1	109	No	Normal	None
		1:145522804	*PEX11B* (NM_003846.3)							
51	M	19: 5151508	*KDM4B* (NM_015015.3)	CAS, receptive-expressive language impairment, normal non-verbal IQ	5	3	91	No	Normal	Drooling up to 4 years
52	M	7:147600801	*CNTNAP2* (NM_014141.6)	CAS, receptive- expressive language impairment, normal non-verbal IQ	5	4	113	No	Normal	None
		1: 6155616	*KCNAB2* (NM_001199862.2)							
53	M	9:98248069	*PTCH1* (NM_000264.5)	CAS, expressive language impairment, normal non-verbal IQ	5	2	93	No	“low” cerebellar amygdalae (but no Chiari 1)	None
54	M	0: 90176938-GCTCATTTCTGC-G	*KIF7* (NM_198525.3)	CAS, receptive- expressive language impairment, normal non-verbal IQ	5	3	91	No	Normal	Twin pregnancy, his sister is healthy.
		9: 19423859	*ACER2* (NM_001010887.3)							
55	F	11:45957270	*PHF21A* (NM_001352027.3)	CAS, expressive language impairment, normal non-verbal IQ	5	2	104	Yes	Normal	None
		17: 8383527	*MYH10* (NM_001256012.3)							
56	M	10:76789416	*KAT6B* (NM_012330.4)	CAS, expressive language impairment, normal non-verbal IQ	5	2	127	No	“low” cerebellar amygdalae (but no Chiari 1)	None
		22: 40060756	*CACNA1I* (ENST00000402142.3)							
57	M	22: 42606366	*TCF20* (NM_001378418.1)	CAS, receptive-expressive language impairment, borderline non-verbal IQ	4	4	74	Yes	Mild hypoplasia of the cerebellar vermis and slight enlargement of the cisterna magna	At birth small for gestational age (3^rd^ percentile). Celiac disease, atopic dermatitis, hypotelorism, depressed eyelid fissures, short philtrum, mild ligament hyperlaxity
58	M	X: 64734848	*LAS1L* (NM_031206.7)	CAS, expressive language impairment, normal non-verbal IQ	5	2	97	No	Normal	None
		2: 54876909	*SPTBN1* (NM_003128.3)							
59	M	2:191797566	*GLS* (NM_001256310.2)	CAS, expressive language impairment, borderline non-verbal IQ	5	1	127	No	Normal	None

*Speech composite severity Score: range 0 (no impairment) – 5 (severe impairment across all the speech measures). Each impaired speech measure (consonantal phonetic repertoire, inaccuracy, inconsistency, syllable omissions, diadochokinetic rate) contributes one point to the composite score.

**Language composite severity score: range 0 (no impairment) – 4 (severe impairment across all the language measures). Each impaired language measure (expressive and receptive lexicon, expressive and receptive grammar) contributes one point to the composite score.

### 3.1 HC: distribution of variants and associated genes

Figure 1A shows the distribution of the variant types, levels of zygosity, and family segregation patterns, including the percentage of *de novo* variants (6/7, 86%), found in the patients with HC variants. Supplementary Table 3A provides the CAS speech features of HC genes.

HC variants occurred in genes already associated with language disorders. In particular, variants were detected in the following genes: *KAT6A* (Zwaveling-Soonawala et al., 2017), *CREBBP* (Menke et al., 2016) and *FGFR3* (Aravidis et al., 2014; Kruszka et al., 2016; Kruszka et al., 1993; Robin et al., 1998). Interestingly, in proband #32 we identified the reported c.749C > G (p.Pro250Arg) variant in *FGFR3* that has been associated, in multiple cases, with Muenke syndrome (MIM: 602849), a condition characterized by considerable phenotypic variability (Kruszka et al., 1993). About 40% of individuals with Muenke syndrome present ID, and 66.3% show developmental delay; some patients, however, as in the case of our proband #32, lack the typical clinical or radiographic features of Muenke syndrome (Kruszka et al., 2016; Kruszka et al., 1993; Robin et al., 1998; Moko and Blandin de Chalain, 2001).

Seven of our CAS probands harbored HC variants — specifically, LoF variants (two frameshift, two non-sense, and one splicing) and two missense variants in putatively novel CAS genes, not yet associated with impaired language phenotypes.

In proband #33, we identified a c.4124del *de novo* frameshift in *ASH1L* regarded as “likely pathogenic.” This gene plays a critical role in development by activating homeobox (HOX) genes and it is associated with autosomal dominant intellectual disability (MIM: 617796) (Okamoto et al., 2017; Wang et al., 2016; Stessman et al., 2017). Table 1 shows the remaining HC variants detected in novel genes.

### 3.2 LC variants: distribution of variants and associated genes

LC variants were identified in 26 children who underwent WES analysis (76% of the sample) (Table 2). These variants are currently classified as class 3 or 4 (see Figure 1B for variant types and zygosity). For variants in genes that do not have a recognized gene-disease association according to OMIM, it was not possible to apply the ACMG classification criteria. As a result, we have annotated these cases in the table as ‘no known gene-disease association’ rather than assigning an ACMG classification. Ten children had more than one variant involving different genes. The Supplementary Table 3B provides the CAS speech features of LC genes.

In the LC subgroup, we identified variants in 33 genes associated with syndromic disorders or other complex neurodevelopmental disorders whose features include speech delay or LI. Fourteen of these genes are listed in the SFARI gene database (Table 2).

Most of the variants were inherited from an unaffected parent (21/34), whereas 13 variants were inherited from a parent reporting early developmental language and speech delay, not otherwise characterized. In 4/38 (11%) cases, variants occurred *de novo* (Figure 1B). Other variants of note included a *de novo* missense variant in *DEPDC5* found in proband #38. LoF variants in *DEPDC5* have been reported in patients with dominantly inherited familial focal epilepsy with variable foci (Samanta, 2022). We also found a *de novo* LC variant in *TOP2B* in case #43. *TOP2B* codes for topoisomerase II isoenzyme beta, which is abundant in both the developing and the adult brain. Defects of topoisomerase can cause developmental delay, ID, hypotonia, microcephaly, and autistic features (Lam et al., 2017; Hiraide et al., 2020). In proband #44 we identified three variants in *SATB2, CSMD1*, and *DCHS1*. Both *SATB2* and *CSMD1* are SFARI genes with a score of 2, and in our study were transmitted by an affected mother and an unaffected father, respectively. Alongside variants in *CSMD1* and *DCHS1* recurred in different CAS probands; indeed, it is interesting to note that in another CAS proband (#30), we identified a splicing de-novo variant in the *CSMD1* gene, highlighting a potential role of this gene in the CAS phenotype. An ultrarare c.214-7C>T variant in HTR3B was identified in two different CAS cases (#46 and #47). Although its effects on splicing are uncertain (see Supplementary Table 4), we evaluated the recurrence of the variant c.214-7C > T in the *HTR3B* gene in our CAS cohort, compared to control individuals. Additional LC genes are listed in Table 2. Also interesting is the involvement of the *TBK1* gene, though there is no evidence of a child neurodevelopmental phenotype associated with this variant. Likely pathogenic c.1330dup (p. Arg444ThrfsTer5) variant in *TBK1* was detected in proband #29, in whom CAS was associated with receptive-expressive LI, and borderline non-verbal IQ. Pathogenic variants in *TBK1* have previously been associated with a combined frontotemporal dementia-amyotrophic lateral sclerosis syndrome, a neurodegenerative condition characterized by adult-onset cognitive decline, behavioral abnormalities, dysarthria, and upper and lower motor neuron involvement. Similarly, a nonsense variant in *TBK1* has already been described in a patient with dysarthria and an asymmetric akinetic-rigid syndrome (Lamb et al., 2019). Although pathogenic variants in *TBK1* have previously been associated with frontotemporal dementia and amyotrophic lateral sclerosis, the phenotype is highly variable, with some cases reported to show primary progressive aphasia and impaired speech production without any other cognitive symptoms (Swift et al., 2021; Price, 2012).

Among the numerous genes considered in this study, some have previously been suggested to be related to CAS. In our study, we identified various variants in genes associated with CAS in the literature, both for HC and LC genes. For example, in two probands (cases #39 and #40), c.440A > G (p.Asp147Gly) and c.3109G > T (p.Asp1037Tyr) were identified in *UBA6*, a known CAS gene (Hildebrand et al., 2020). In case #41, c.74C > G (p. Thr25Arg) was found in *ZFHX4*, which others have already shown to be involved (Kaspi et al., 2023; Eising et al., 2019). Also, HC variant identified in *KAT6A* has already been described as CAS-associated variants (Eising et al., 2019; Kaspi et al., 2023). A gene enrichment analysis (Supplementary Figure 3; Supplementary Table 5), performed by sequencing data drawn from the “1,000 Genomes” database and referring to 107 healthy Italians (Tuscany) plus 395 healthy Europeans of multiple genetic backgrounds (CEU, FIN, GBR, IBS), revealed that rare variants (frequency < 1%) in recurrent genes *UBA6*, *ZFHX4*, and *KAT6A* are more common in children with CAS.

### 3.3 Gene ontology and expression profile of CAS genes

**Figure 2:**
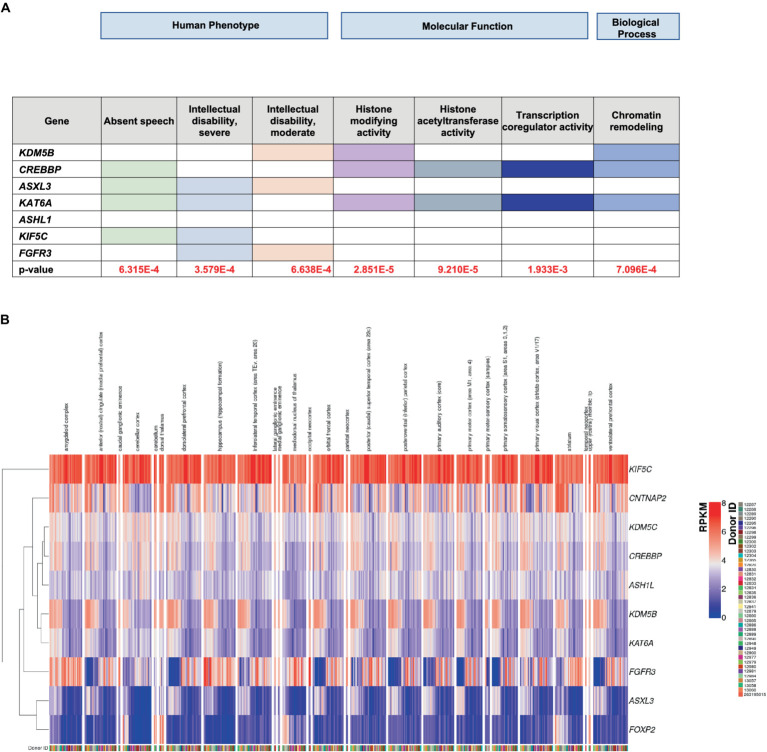
**(A)** ToppGene enrichment analysis of the GO human phenotype ontology, molecular function and biological process. **(B)** RNA-seq RPKM values obtained from the BrainSpan database for the set of high-confidence (HC) genes. For each donor available in the BrainSpan Atlas of the Developing Human Brain database, the RNA-seq expression values of the HC genes are plotted using the ComplexHeatmap 2 R package. The heatmap is divided into submodules, each representing the genes expression values for a particular brain area, described at the top of the plot. Note that each donor is associated with one or more columns, since the RPKM values are available for multiple brain areas. Genes are also clustered based on their RPKM values, as represented by the dendogram on the left, in order to better show expression similarity between genes.

In an effort to provide a biological interpretation of our data, we performed Gene Ontology (GO) analysis of all the HC genes, using the TOPPGENE database. “Absent speech,” “Intellectual disability, severe” and “Intellectual disability, moderate” (Figure 2A) were found to be the prevalent HPO terms associated with candidate HC genes. GO analysis of the molecular functions, in which the HC genes are involved, suggested the presence of epigenetic mechanisms (Figure 2A). Indeed, these genes encode for DNA-binding proteins and may act as transcriptional activators or suppressors by modulating the expression of target genes. Most HC genes also participate in biological processes involved in chromatin remodeling (Figure 2A), a mechanism closely related to neurodevelopment. Finally, using the BrainSpan database,^6^ we were able to derive the spatial and temporal expressions of both the HC and the LC genes found in this study and relate them to different brain areas. Preliminary observation, based on the visual analysis of color intensities of the gene enrichment analysis heatmap (Figure 2B). (Figure 2B) showed that *KIF5C* is highly expressed (red) in many brain regions, indicating strong expression in these tissues; three HC genes (*CREBBP*, *ASHL1* and *KDM5B*), in addition to four LC ones (*DDX1*, *PQBP1*, *MYH10*, *PEX11B*) (Supplementary Figure 4), were highly co-expressed throughout the brain, although the cerebellum and dorsal thalamus were the most involved areas. Both of these brain areas contribute to speech and language processing in normal and pathological conditions (Silveri, 2021; Price, 2012; Klostermann et al., 2013).

## 4 Discussion

This single-center study performed in Italian children to describe the expanding molecular landscape of CAS yielded potentially relevant findings.

### 4.1 Molecular characterization and findings

First, molecular characterization was achieved in 21.35% of the cohort, a diagnostic yield that is lower than those found by others using WES. Hildebrand and coworkers obtained a diagnostic rate of 32% (Hildebrand et al., 2020), Eising and collaborators (Eising et al., 2019) a diagnostic rate of 42% and Kaspi and colleagues (Eising et al., 2019) recorded a 26% rate. These findings, however, show that this group of Italian CAS children, like other cohorts, is well characterized, with the identification of some causal genes confirming the remarkable genetic heterogeneity of this disorder. Our findings are consistent with those of previous studies, including a recent review by Morgan et al. (2024), which identified significant genetic heterogeneity in CAS with many implicated genes converging on pathways involved in chromatin modification and transcriptional regulation (Morgan et al., 2024). This reinforces the importance of these pathways in the etiology of CAS and supports the potential for precision diagnosis and targeted therapies.

Second, we described a number of new pathogenic/likely pathogenic variants and novel potential CAS genes, and defined a network that includes both new and previously reported CAS genes. Moreover, we aimed to enhance our understanding of genotype–phenotype correlations. The integration of new findings allows genetic characterization and more precise phenotype identification in most children.

Unlike our earlier study (Chilosi et al., 2022), the present research focused on children with CAS and no co-occurrent ID, ASD and ADHD. Using WES, we found both HC (7 children) and LC (26 children) variants.

Third, all the HC genes found in this study are involved in processes underlying speech and language acquisition, and their alterations yield phenotypes characterized by delayed expressive language, poor or absent speech, LI and ID.

### 4.2 HC genes, haploinsufficiency, and clinical relevance

Our findings highlight a role for pathogenic/likely pathogenic variants in genes that have either already been related to speech and language impairment (namely, *KAT6A*, *CREBBP* and *FGFR3*), or can be recognized as new possible players, closely linked to neurodevelopmental disorders. It is also of note that our CAS cohort was enriched in variants occurring in genes that encode proteins involved in epigenetic mechanisms and are expressed in brain regions closely linked to language acquisition processes (Silveri, 2021; Price, 2012; Klostermann et al., 2013).

Although HC genes were selected based on their known involvement in language and neurodevelopment, the identification of specific variants provides new insights into their role in CAS. Indeed, HC variants in *ASH1L* and *KDM5B* were first reported in relation to CAS phenotypes. In line with what is known in the literature for *ASH1L*, where loss-of-function variants predominate in intellectual disability, we also identified variants causing premature termination codons in our probands. This is consistent with previous studies that have shown a high prevalence of PTVs in dominant developmental disorders (Faundes et al., 2018).

*ASH1L* and *KDM5B* are genes that encode components of the epigenetic apparatus (Miyazaki et al., 2013; Shao et al., 2014), and the superimposed cognitive features such as speech/language deficits that have been reported in chromatin-related disorders (Ng et al., 2023), seem to indicate molecular effects on neural systems dedicated to language and oromotor functioning. Heterozygous variants in *ASH1L* have been identified in patients with autosomal dominant ID, absent speech, and behavioral abnormalities (OMIM: 617796) (Okamoto et al., 2017; Wang et al., 2016; Stessman et al., 2017). This gene plays a critical role in neurodevelopment by activating HOX genes. It catalyzes mono- and demethylation of H3K36, promoting gene expression (Miyazaki et al., 2013), and it is highly expressed in both embryonic and adult brains (Okamoto et al., 2017). *KDM5B* was first related to autosomal recessive ID in 2018 (Faundes et al., 2018), however scientific evidence suggested that, unlike homozygous LoF variants, which are likely fully penetrant, heterozygous LoF variants of the same gene have incomplete penetrance and are implicated in autosomal dominant ID and ASD (Borroto et al., 2024.).

As in other neurodevelopmental conditions, the detection of pathogenic or likely pathogenic variants is crucial to allow early identification and prioritization of cases needing further in-depth clinical assessment. Our proband #27, for example, harbored a *de novo* frameshift alteration in *KAT6A*, a relatively rare etiology in CAS, ASD, ID, and severe language and communication disorders (Kennedy et al., 2019; St John et al., 2022). Alongside complex and severe presentations, some alterations in *KAT6A* can also show milder profiles and minor behavioral symptoms (St John et al., 2022). Our findings are in line with those of Kennedy et al., (Kennedy et al., 2019) who observed greater severity of developmental delay and an increased frequency associated with truncating pathogenic variants in the last two exons, suggesting that nonsense-mediated decay may play a role in mitigating the phenotype. Nevertheless, the presence of other clinical features — our case, for example, showed strabismus, early feeding difficulties, and hypotonia, associated with markedly delayed and effortful acquisition of speech and the absence of microcephaly, cardiac abnormalities, and gastrointestinal complications — can facilitate earlier molecular identification. Moreover, detailed neuropsychological phenotyping, performed in a longitudinal perspective, may provide precious information on the long-term outcome of CAS, making it possible to hypothesize a grading of clinical severity based on the deleteriousness of the gene variant. As an example, proband #41 harbored a missense variant in *ZFHX4*, a gene in which LoF variants usually result in a complex neuropsychological phenotype characterized by developmental delay, ID, and motor speech and language impairment (Fontana et al., 2021). Long-term follow up of this case allowed us to rule out cognitive difficulties, to track changes in language acquisition up to normal functioning, but also to detect the persistence of the motor speech disorder and the emergence of academic learning difficulties at school age.

In our work, the use of dataset enrichment to interpret clinical phenotype is further corroborated by the evidence concerning the LC genes, of which 18/38 (namely, *SATB2, SRPX2, LAS1L, SPTBN1, GLS, ZNF142, TCF20, SCN8A, CNTNAP2, KCNAB2, MACF1, CACNA1I, ASXL2, PTCH1, DLL1, KAT6B, PHF21A, KDM4B*) are involved in delayed speech and language development (HP:0000750, *p* = 6.433E-8), language impairment (HP:0002463, *p* = 9.713E-9), and feeding difficulties (HP:0011968. *p* = 1.531E-6). Additionally, 11/38 LC genes (*ZNF142, LASL1, TCF20, SCN8A, MACF1, PEX11B, DLL1, PQBP1, PHF21A, KIF7, SCN8A*) are significantly associated with ID (DisGeNET database, *p* = 3.933E-11). It is tempting to speculate that minor genetic hits, such as those resulting from LC variants, have some effects during development and influence variable clinical expressivity linked to other factors, including non-genetic ones.

### 4.3 Gene expression, enrichment analysis, and brain structures involvement

Finally, our attempt to correlate novel etiologies in CAS with the relative genes’ molecular function and spatio-temporal expression in the brain seemed to suggest a role in CAS for two brain structures (cerebellum and dorsal thalamus), both highly involved in neurodevelopment and speech acquisition. A theoretical model of speech motor control acquisition, the DIVA model, maintains that the cerebellum plays a crucial role in the generation of feed-forward motor commands for speech, thus allowing the efficient production of pre-programmed and pre-planned movement sequences. Children with CAS are hypothesized to be unable to generate accurate anticipatory motor commands, and to therefore retain an immature control strategy that relies on slow, retroactive mechanisms (Terband et al., 2009). We ourselves (Fiori et al., 2016), using a connectome approach to study the brains of children with CAS, found reduced connectivity in three subnetworks, one of which (subnetwork 2) also includes the right cerebellum. Finally, a body of literature reports a wide range of symptoms related to speech, language, executive functioning, visuo-spatial processing, academic learning, and behavioral disturbances in children with cerebellar dysfunctions, either acquired or congenital (Riva and Giorgi, 2000; Koziol et al., 2014; Salman and Tsai, 2016; Panagopoulos et al., 2022). The thalamus and the higher-order dorsal thalamic nuclei in particular are richly connected to the prefrontal motor cortices, whose role in speech generation and motor control is well known. Interestingly, the dorsal thalamus appears to play a role in informing several cortical areas of an upcoming motor command and in providing an anticipatory model of the expected sensory consequences of a motor response (Sherman, 2007). Beyond the suggested implications of the dorsal thalamus in motor control, some authors also highlight its functional contributions to distinct cognitive and executive skills, such as working memory, new learning, adaptive decision making, attention and language (Ouhaz et al., 2018). Relationships between speech, language and thalamic function have also been studied after thalamotomy in children (Nass et al., 2000). The role of the cerebellum in generating motor commands for speech and the thalamus’s function in motor and cognitive control further highlight their importance in CAS.

Deep speech and language phenotyping revealed a different profile between the HC and LC groups. With regard to the severity of the speech disorder, our comparison of these two groups did not reveal any statistically significant difference, but the number of children with receptive-expressive language impairment was higher among those with HC variants. This finding is of clinical relevance. If confirmed in other study cohorts, and for speakers of other languages, it should inform therapeutic choices and allow a better approach to the comorbid manifestations that can be present in children with CAS (Miller et al., 2019). Although additional studies are necessary to consolidate this information, our expansion of the genetic landscape in CAS allowed us to focus on molecular pathways and regional and anatomical brain expression. This approach could potentially help to disentangle the complex mechanisms leading to impaired language acquisition processes, and favor the development of new tools for early detection and better care. Given the genetic heterogeneity and pleiotropy, the assessment of children with CAS should be multidisciplinary, as some genetic variants could be associated not only to other neurodevelopmental disorders, but also to multiple organs alterations, such as eye defects (i.e., in *KAT6A*). Moreover, the frequent association of CAS with language disorder, the increased risk of developing academic learning difficulties and atypical implicit learning (Bombonato et al., 2022), mandates intensive therapy and educational management aimed to provide specific and dynamic support to the children and to their families across the different needs emerging in development. Alternative strategies for communications should be implemented to support children with the most severe, persistent and complex presentations of the disorder. Deep speech, language and cognitive phenotyping is crucial for future genetic research and for clinical decision making.

## Data Availability

The data presented in the study are deposited in the European Variation Archive (EVA) at EMBL-EBI, accession number PRJEB74924 (https://www.ebi.ac.uk/eva/?eva-study=PRJEB74924).

## Footnotes

^1^ https://www.asha.org/policy/tr2007-00278/

^2^https://broadinstitute.github.io/picard/ accessed on 14 December 2023

^3^https://www.sfari.org/ last accessed on December 31, 2022

^4^ https://www.brainspan.org/rnaseq/searches

^5^ http://bioconductor.org/packages/release/bioc/html/ComplexHeatmap.html

^6^ https://www.brainspan.org

## References

[ref1] American Speech-Language-Hearing Association. (2007). Childhood apraxia of speech [technical report]. Available at: https://www.asha.org/policy/tr2007-00278

[ref2] Aravidis C. Konialis C. P. Pangalos C. G. Kosmaidou Z. (2014). A familial case of Muenke syndrome. Diverse expressivity of the FGFR3 Pro252Arg mutation--case report and review of the literature. J. Matern. Fetal Neonatal Med. 27, 1502–1506. doi: 10.3109/14767058.2013.860520 24168007

[ref3] Bombonato C. Casalini C. Pecini C. Angelucci G. Vicari S. Podda I.. (2022). Implicit learning in children with childhood apraxia of speech. Res. Dev. Disabil. 122:104170. doi: 10.1016/j.ridd.2021.104170, PMID: 35030467

[ref4] Borroto M. C. Michaud C. Hudon C. Agrawal P. B. Agre K. Applegate C. D.. (2024). A genotype/phenotype study of *KDM5B*-associated disorders suggests a pathogenic effect of dominantly inherited missense variants. Genes 15:1033. doi: 10.3390/genes15081033 39202393 PMC11353349

[ref5] Cassar C. McCabe P. Cumming S. (2023). “I still have issues with pronunciation of words”: a mixed methods investigation of the psychosocial and speech effects of childhood apraxia of speech in adults. Int. J. Speech Lang. Pathol. 25, 193–205. doi: 10.1080/17549507.2021.2018496 35034534

[ref6] Chenausky K. V. Baas B. Stoeckel R. Brown T. Green J. R. Runke C.. (2023). Comorbidity and severity in childhood apraxia of speech: a retrospective chart review. J. Speech Lang. Hear. Res. 66, 1–13. doi: 10.1044/2022_JSLHR-22-00436, PMID: 36795544 PMC10205100

[ref7] Chenausky K. V. Brignell A. Morgan A. Gagné D. Norton A. Tager-Flusberg H.. (2020). Factor analysis of signs of childhood apraxia of speech. J. Commun. Disord. 87:106033. doi: 10.1016/j.jcomdis.2020.106033 32877838 PMC7494519

[ref8] Chilosi A. M. Brovedani P. Cipriani P. Casalini C. (2023). Sex differences in early language delay and in developmental language disorder. J. Neurosci. Res. 101, 654–667. doi: 10.1002/jnr.24976 34822733

[ref9] Chilosi A. M. Lorenzini I. Fiori S. Graziosi V. Rossi G. Pasquariello R.. (2015). Behavioral and neurobiological correlates of childhood apraxia of speech in Italian children. Brain Lang. 150, 177–185. doi: 10.1016/j.bandl.2015.10.002, PMID: 26552038

[ref10] Chilosi A. M. Pfanner L. Pecini C. Salvadorini R. Casalini C. Brizzolara D.. (2019). Which linguistic measures distinguish transient from persistent language problems in late talkers from 2 to 4 years? A study on Italian speaking children. Res. Dev. Disabil. 89, 59–68. doi: 10.1016/j.ridd.2019.03.005 30947105

[ref11] Chilosi A. M. Podda I. Ricca I. Comparini A. Franchi B. Fiori S.. (2022). Differences and commonalities in children with childhood apraxia of speech and comorbid neurodevelopmental disorders: a multidimensional perspective. J Pers Med 12:313. doi: 10.3390/jpm12020313, PMID: 35207801 PMC8880782

[ref12] Conti E. Retico A. Palumbo L. Spera G. Bosco P. Biagi L.. (2020). Autism Spectrum disorder and childhood apraxia of speech: early language-related hallmarks across structural MRI study. J Pers Med 10:275. doi: 10.3390/jpm10040275, PMID: 33322765 PMC7768516

[ref13] Eising E. Carrion-Castillo A. Vino A. Strand E. A. Jakielski K. J. Scerri T. S.. (2019). A set of regulatory genes co-expressed in embryonic human brain is implicated in disrupted speech development. Mol. Psychiatry 24, 1065–1078. doi: 10.1038/s41380-018-0020-x, PMID: 29463886 PMC6756287

[ref14] Faundes V. Newman W. G. Bernardini L. Canham N. Clayton-Smith J. Dallapiccola B.. (2018). Histone lysine methylases and demethylases in the landscape of human developmental disorders. Am. J. Hum. Genet. 102, 175–187. doi: 10.1016/j.ajhg.2017.11.013, PMID: 29276005 PMC5778085

[ref15] Fiori S. Guzzetta A. Mitra J. Pannek K. Pasquariello R. Cipriani P.. (2016). Neuroanatomical correlates of childhood apraxia of speech: a connectomic approach. Neuroimage Clin 12, 894–901. doi: 10.1016/j.nicl.2016.11.003, PMID: 27882295 PMC5114583

[ref16] Fontana P. Ginevrino M. Bejo K. Cantalupo G. Ciavarella M. Lombardi C.. (2021). A ZFHX4 mutation associated with a recognizable neuropsychological and facial phenotype. Eur. J. Med. Genet. 64:104321. doi: 10.1016/j.ejmg.2021.104321, PMID: 34461323

[ref17] Hall P. K. Jordan L. S. Robin D. A. (1993). Developmental apraxia of speech: Theory and clinical practice. Virginia: Pro-Ed, Università della.

[ref18] Hildebrand M. S. Jackson V. E. Scerri T. S. Van Reyk O. Coleman M. Braden R. O.. (2020). Severe childhood speech disorder: gene discovery highlights transcriptional dysregulation. Neurology 94, e2148–e2167. doi: 10.1212/WNL.0000000000009441, PMID: 32345733

[ref19] Hiraide T. Watanabe S. Matsubayashi T. Yanagi K. Nakashima M. Ogata T.. (2020). A de novo TOP2B variant associated with global developmental delay and autism spectrum disorder. Mol. Genet. Genomic Med. 8:e1145. doi: 10.1002/mgg3.1145 31953910 PMC7057084

[ref20] Iuzzini-Seigel J. Allison K. M. Stoeckel R. (2022). A tool for differential diagnosis of childhood apraxia of speech and dysarthria in children: a tutorial. Lang. Speech Hear. Serv. Sch. 53, 926–946. doi: 10.1044/2022_LSHSS-21-00164, PMID: 35523425

[ref21] Kadis D. S. Goshulak D. Namasivayam A. Pukonen M. Kroll R. De Nil L. F.. (2014). Cortical thickness in children receiving intensive therapy for idiopathic apraxia of speech. Brain Topogr. 27, 240–247. doi: 10.1007/s10548-013-0308-8, PMID: 23974724 PMC3921462

[ref22] Kaspi A. Hildebrand M. S. Jackson V. E. Braden R. van Reyk O. Howell T.. (2023). Genetic aetiologies for childhood speech disorder: novel pathways co-expressed during brain development. Mol. Psychiatry 28, 1647–1663. doi: 10.1038/s41380-022-01764-8, PMID: 36117209 PMC10208970

[ref23] Kennedy J. Goudie D. Blair E. Chandler K. Joss S. McKay V.. (2019). KAT6A syndrome: genotype-phenotype correlation in 76 patients with pathogenic KAT6A variants. Genet. Med. 21, 850–860. doi: 10.1038/s41436-018-0259-2, PMID: 30245513 PMC6634310

[ref24] Klostermann F. Krugel L. K. Ehlen F. (2013). Functional roles of the thalamus for language capacities. Front. Syst. Neurosci. 7:32. doi: 10.3389/fnsys.2013.00032 23882191 PMC3712252

[ref25] Koziol L. F. Budding D. Andreasen N. D’Arrigo S. Bulgheroni S. Imamizu H.. (2014). Consensus paper: the cerebellum’s role in movement and cognition. Cerebellum 13, 151–177. doi: 10.1007/s12311-013-0511-x, PMID: 23996631 PMC4089997

[ref26] Kruszka P. Addissie Y. A. Yarnell C. M. Hadley D. W. Guillen Sacoto M. J. Platte P.. (2016). Muenke syndrome: an international multicenter natural history study. Am. J. Med. Genet. A 170, 918–929. doi: 10.1002/ajmg.a.37528 26740388

[ref27] Kruszka P. Rolle M. Kahle K. T. Muenke M. (1993). “Muenke Syndrome” in GeneReviews((R)). eds. Adam M. P. Feldman J. Mirzaa G. M. Pagon R. A. Wallace S. E. Bean L. J. H.. (Seattle (WA): University of Washington). 20301588

[ref28] Lam C. W. Yeung W. L. Law C. Y. (2017). Global developmental delay and intellectual disability associated with a de novo TOP2B mutation. Clin. Chim. Acta 469, 63–68. doi: 10.1016/j.cca.2017.03.022 28343847

[ref29] Lamb R. Rohrer J. D. Real R. Lubbe S. J. Waite A. J. Blake D. J.. (2019). A novel TBK1 mutation in a family with diverse frontotemporal dementia spectrum disorders. Cold Spring Harb Mol Case Stud 5:a003913. doi: 10.1101/mcs.a003913, PMID: 31160356 PMC6549548

[ref31] Lewis B. A. Benchek P. Tag J. Miller G. Freebairn L. Taylor H. G.. (2021). Psychosocial comorbidities in adolescents with histories of childhood apraxia of speech. Am. J. Speech Lang. Pathol. 30, 2572–2588. doi: 10.1044/2021_AJSLP-21-00035, PMID: 34609155 PMC9132062

[ref32] Lewis B. A. Freebairn L. A. Hansen A. J. Iyengar S. K. Taylor H. G. (2004). School-age follow-up of children with childhood apraxia of speech. Lang. Speech Hear. Serv. Sch. 35, 122–140. doi: 10.1044/0161-1461(2004/014) 15191325

[ref33] Lewis B. A. Miller G. J. Iyengar S. K. Stein C. Benchek P. (2023). Long-term outcomes for individuals with childhood apraxia of speech. J. Speech Lang. Hear. Res. 1–17. doi: 10.1044/2023_JSLHR-22-00647 37734154

[ref34] Li H. Handsaker B. Wysoker A. Fennell T. Ruan J. Homer N.. (2009). The sequence alignment/map format and SAMtools. Bioinformatics 25, 2078–2079. doi: 10.1093/bioinformatics/btp352, PMID: 19505943 PMC2723002

[ref30] Maleki F. Ovens K. Hogan D. J. Kusalik A. J. (2020). Gene Set Analysis: Challenges, Opportunities, and Future Research. Front. Genet. 11:654. doi: 10.3389/fgene.2020.00654 32695141 PMC7339292

[ref35] Menke L. A. van Belzen M. J. Alders M. Cristofoli F. Ehmke N. Fergelot P.. (2016). CREBBP mutations in individuals without Rubinstein-Taybi syndrome phenotype. Am. J. Med. Genet. A 170, 2681–2693. doi: 10.1002/ajmg.a.37800 27311832

[ref36] Miller G. J. Lewis B. Benchek P. Freebairn L. Tag J. Budge K.. (2019). Reading outcomes for individuals with histories of suspected childhood apraxia of speech. Am. J. Speech Lang. Pathol. 28, 1432–1447. doi: 10.1044/2019_AJSLP-18-0132, PMID: 31419159 PMC7251600

[ref37] Miller G. J. Lewis B. A. (2022). Reading skills in children with suspected childhood apraxia of speech and children with Reading disorders: same or different? Lang. Speech Hear. Serv. Sch. 53, 985–1005. doi: 10.1044/2022_LSHSS-21-00149 35947819

[ref38] Miller J. A. Ding S. L. Sunkin S. M. Smith K. A. Ng L. Szafer A.. (2014). Transcriptional landscape of the prenatal human brain. Nature 508, 199–206. doi: 10.1038/nature13185, PMID: 24695229 PMC4105188

[ref39] Miyazaki H. Higashimoto K. Yada Y. Endo T. A. Sharif J. Komori T.. (2013). Ash1l methylates Lys36 of histone H3 independently of transcriptional elongation to counteract polycomb silencing. PLoS Genet. 9:e1003897. doi: 10.1371/journal.pgen.1003897, PMID: 24244179 PMC3820749

[ref40] Moko S. B. Blandin de Chalain T. M. (2001). New Zealand Maori family with the pro250arg fibroblast growth factor receptor 3 mutation associated with craniosynostosis. J. Craniomaxillofac. Surg. 29, 22–24. 11467490

[ref41] Morgan A. Amor J. D. John M. D. Sheffer I. E. Hildebrand M. S. (2024). Genetic architecture of childhood speech disorder: a review. Mol. Psychiatry 29, 1281–1292. doi: 10.1038/s41380-024-02409-8, PMID: 38366112 PMC11189821

[ref42] Morison L. D. Meffert E. Stampfer M. Wilke I. S. Vollmer B. V. Schulze K.. (2023). In-depth characterisation of a cohort of individuals with missense and loss-of-function variants disrupting FOXP2. J. Med. Genet. 60, 597–607. doi: 10.1136/jmg-2022-108734, PMID: 36328423 PMC10314088

[ref43] Murray E. Thomas D. McKechnie J. (2019). Comorbid morphological disorder apparent in some children aged 4-5 years with childhood apraxia of speech: findings from standardised testing. Clin. Linguist. Phon. 33, 42–59. doi: 10.1080/02699206.2018.1513565 30199280

[ref44] Nass R. Boyce L. Leventhal F. Levine B. Allen J. Maxfield C.. (2000). Acquired aphasia in children after surgical resection of left-thalamic tumors. Dev. Med. Child Neurol. 42, 580–590. doi: 10.1017/s0012162200001109, PMID: 11034450

[ref45] Ng R. Harris J. Kleefstra T. Morgan A. T. Simpson B. (2023). Editorial: characterizing the neurobehavioral phenotype of mendelian disorders of epigenetic machinery. Front. Genet. 14:1338078. doi: 10.3389/fgene.2023.1338078 38116293 PMC10728862

[ref46] Okamoto N. Miya F. Tsunoda T. Kato M. Saitoh S. Yamasaki M.. (2017). Novel MCA/ID syndrome with ASH1L mutation. Am. J. Med. Genet. A 173, 1644–1648. doi: 10.1002/ajmg.a.38193, PMID: 28394464

[ref47] Ouhaz Z. Fleming H. Mitchell A. S. (2018). Cognitive functions and neurodevelopmental disorders involving the prefrontal cortex and mediodorsal thalamus. Front. Neurosci. 12:33. doi: 10.3389/fnins.2018.00033 29467603 PMC5808198

[ref48] Panagopoulos D. Stranjalis G. Gavra M. Boviatsis E. Korfias S. Karydakis P.. (2022). The entity of cerebellar mutism syndrome: a narrative review centered on the etiology, diagnostics, prevention, and therapeutic options. Children 10:83. doi: 10.3390/children10010083, PMID: 36670634 PMC9856273

[ref49] Price C. J. (2012). A review and synthesis of the first 20 years of PET and fMRI studies of heard speech, spoken language and reading. NeuroImage 62, 816–847. doi: 10.1016/j.neuroimage.2012.04.062 22584224 PMC3398395

[ref50] Richards S. Aziz N. Bale S. Bick D. Das S. Gastier-Foster J.. (2015). Standards and guidelines for the interpretation of sequence variants: a joint consensus recommendation of the American College of Medical Genetics and Genomics and the Association for Molecular Pathology. Genet. Med. 17, 405–424. doi: 10.1038/gim.2015.30, PMID: 25741868 PMC4544753

[ref51] Riva D. Giorgi C. (2000). The cerebellum contributes to higher functions during development: evidence from a series of children surgically treated for posterior fossa tumours. Brain 123, 1051–1061. doi: 10.1093/brain/123.5.1051 10775549

[ref52] Robin N. H. Scott J. A. Cohen A. R. Goldstein J. A. (1998). Nonpenetrance in FGFR3-associated coronal synostosis syndrome. Am. J. Med. Genet. 80, 296–297. doi: 10.1002/(SICI)1096-8628(19981116)80:3<296::AID-AJMG25>3.0.CO;2-6 9843059

[ref53] Salman M. S. Tsai P. (2016). The role of the pediatric cerebellum in motor functions, cognition, and behavior: a clinical perspective. Neuroimaging Clin. N. Am. 26, 317–329. doi: 10.1016/j.nic.2016.03.003, PMID: 27423796 PMC4948592

[ref54] Samanta D. (2022). DEPDC5-related epilepsy: a comprehensive review. Epilepsy Behav. 130:108678. doi: 10.1016/j.yebeh.2022.108678, PMID: 35429726

[ref55] Shao G. B. Chen J. C. Zhang L. P. Huang P. Lu H. Y. Jin J.. (2014). Dynamic patterns of histone H3 lysine 4 methyltransferases and demethylases during mouse preimplantation development. In Vitro Cell. Dev. Biol. Anim. 50, 603–613. doi: 10.1007/s11626-014-9741-6, PMID: 24619213

[ref56] Sherman S. M. (2007). The thalamus is more than just a relay. Curr. Opin. Neurobiol. 17, 417–422. doi: 10.1016/j.conb.2007.07.003, PMID: 17707635 PMC2753250

[ref57] Shriberg L. D. Aram D. M. Kwiatkowski J. (1997). Developmental apraxia of speech: I. Descriptive and theoretical perspectives. J. Speech Lang. Hear. Res. 40, 273–285. doi: 10.1044/jslhr.4002.273 9130199

[ref58] Shriberg L. D. Kwiatkowski J. Mabie H. L. (2019a). Estimates of the prevalence of motor speech disorders in children with idiopathic speech delay. Clin. Linguist. Phon. 33, 679–706. doi: 10.1080/02699206.2019.1595731 30987467 PMC6633906

[ref59] Shriberg L. D. Potter N. L. Strand E. A. (2011). Prevalence and phenotype of childhood apraxia of speech in youth with galactosemia. J. Speech Lang. Hear. Res. 54, 487–519. doi: 10.1044/1092-4388(2010/10-0068), PMID: 20966389 PMC3070858

[ref60] Shriberg L. D. Strand E. A. Jakielski K. J. Mabie H. L. (2019b). Estimates of the prevalence of speech and motor speech disorders in persons with complex neurodevelopmental disorders. Clin. Linguist. Phon. 33, 707–736. doi: 10.1080/02699206.2019.1595732 31221012 PMC6633911

[ref61] Silveri M. C. (2021). Contribution of the cerebellum and the basal ganglia to language production: speech, word fluency, and sentence construction-evidence from pathology. Cerebellum 20, 282–294. doi: 10.1007/s12311-020-01207-6 33120434 PMC8004516

[ref62] St John M. Amor D. J. Morgan A. T. (2022). Speech and language development and genotype-phenotype correlation in 49 individuals with KAT6A syndrome. Am. J. Med. Genet. A 188, 3389–3400. doi: 10.1002/ajmg.a.62899 35892268

[ref63] Stein C. M. Benchek P. Miller G. Hall N. B. Menon D. Freebairn L.. (2020). Feature-driven classification reveals potential comorbid subtypes within childhood apraxia of speech. BMC Pediatr. 20:519. doi: 10.1186/s12887-020-02421-1, PMID: 33187500 PMC7664029

[ref64] Stessman H. A. Xiong B. Coe B. P. Wang T. Hoekzema K. Fenckova M.. (2017). Targeted sequencing identifies 91 neurodevelopmental-disorder risk genes with autism and developmental-disability biases. Nat. Genet. 49, 515–526. doi: 10.1038/ng.3792 28191889 PMC5374041

[ref65] Swift I. J. Bocchetta M. Benotmane H. Woollacott I. O. Shafei R. Rohrer J. D. (2021). Variable clinical phenotype in TBK1 mutations: case report of a novel mutation causing primary progressive aphasia and review of the literature. Neurobiol. Aging 99, 100 e109–100 e115. doi: 10.1016/j.neurobiolaging.2020.08.014 PMC7907669 32980182

[ref66] Terband H. Maassen B. Guenther F. H. Brumberg J. (2009). Computational neural modeling of speech motor control in childhood apraxia of speech (CAS). J. Speech Lang. Hear. Res. 52, 1595–1609. doi: 10.1044/1092-4388(2009/07-0283), PMID: 19951927 PMC2959199

[ref67] Thorvaldsdottir H. Robinson J. T. Mesirov J. P. (2013). Integrative genomics viewer (IGV): high-performance genomics data visualization and exploration. Brief. Bioinform. 14, 178–192. doi: 10.1093/bib/bbs017 22517427 PMC3603213

[ref68] Wang K. Li M. Hakonarson H. (2010). ANNOVAR: functional annotation of genetic variants from high-throughput sequencing data. Nucleic Acids Res. 38:e164. doi: 10.1093/nar/gkq603 20601685 PMC2938201

[ref69] Wang T. Guo H. Xiong B. Stessman H. A. Wu H. Coe B. P.. (2016). De novo genic mutations among a Chinese autism spectrum disorder cohort. Nat. Commun. 7:13316. doi: 10.1038/ncomms13316, PMID: 27824329 PMC5105161

[ref70] Zhang Y. Wang Q. Zhang Y. Chen Y. Liu T. Zhao J.. (2023). Functional enrichment analysis of mutated genes in neuropsychiatric disorders using TOPPGENE. Neuropsychiatric Genetics 19, 215–230. doi: 10.1093/neuogen/gny123

[ref71] Zwaveling-Soonawala N. Maas S. M. Alders M. Majoie C. B. Fliers E. van Trotsenburg A. S. P.. (2017). Variants in KAT6A and pituitary anomalies. Am. J. Med. Genet. A 173, 2562–2565. doi: 10.1002/ajmg.a.38330 28636259

