# Supplementary material for: Expanding the molecular landscape of childhood apraxia of speech: evidence from a single-center experience
Source: Front Neurosci. 2024 Sep 24;18:1396240. doi: 10.3389/fnins.2024.1396240 (PMC11459770; doi:10.3389/fnins.2024.1396240)
Supplement: Supplementary file 3 [file Table_3.docx]

**Supplementary Table 3a: the CAS speech features presented by the children with High Confidence gene alterations according to ASHA (2007) and to the Mayo checklist.**

| Proband # |  | 27 | 28 | 32 | 33 | 34 | 35 | 37 |
| --- | --- | --- | --- | --- | --- | --- | --- | --- |
| ASHA:  3 speech features | 1. Inconsistent errors on consonants and vowels in repeated productions of syllables or words. | + | + | + | + | + | + | + |
|  | 1. Lengthened and disrupted co-articulatory transitions between sounds and syllables. | + | + | + | + | + | + | + |
|  | 1. Inappropriate prosody, especially in the realization of lexical or phrasal stress. | + | + | + | + | + | + | + |
| Mayo Checklist |  |  |  |  |  |  |  |  |
|  | 1. Vowel or consonant errors including distorted substitutions. | + | + | + | + | + | + | + |
|  | 1. Intrusive *schwa*. | - | - | - | - | - | - | - |
|  | 1. Voicing errors. | + | + | + | - | + | + | + |
|  | 1. Lexical stress errors or equal stress. | + | + | + | - | + | + | + |
|  | 1. Syllable segregation. | + | + | + | - | + | + | + |
|  | 1. Slow rate | + | + | - | - | - | + | + |
|  | 1. Difficulty achieving initial articulatory configurations and transitions into vowels. | + | + | + | + | + | + | + |
|  | 1. Slow DDK rate. | + | + | + | + | + | + | + |
|  | 1. Groping (articulatory searching prior to phonation). | + | + | + | + | + | + | + |
|  | 1. Increased difficulty with longer or more phonetically complex words. | + | + | + | + | + | + | + |

**Supplementary Table 3b: the CAS speech features presented by the children with Low Confidence gene alterations according to ASHA (2007) and to the Mayo checklist.**

| Proband # |  | 29 | 30 | 31 | 36 | 38 | 39 | 40 | 41 | 42 | 43 | 44 | 45 | 46 | 47 | 48 | 49 | 50 | 51 | 52 | 53 | 54 | 55 | 56 | 57 | 58 | 59 |
| --- | --- | --- | --- | --- | --- | --- | --- | --- | --- | --- | --- | --- | --- | --- | --- | --- | --- | --- | --- | --- | --- | --- | --- | --- | --- | --- | --- |
| ASHA:  3 speech features | 1. Inconsistent errors on consonants and vowels in repeated productions of syllables or words. | + | + | + | + | + | + | + | + | + | + | + | + | + | + | + | + | + | + | + | + | + | + | + | + | + | + |
|  | 1. Lengthened and disrupted co-articulatory transitions between sounds and syllables. | + | + | + | + | + | + | + | + | + | + | + | + | + | + | + | + | + | + | + | + | + | + | + | + | + | + |
|  | 1. Inappropriate prosody, especially in the realization of lexical or phrasal stress. | + | + | + | + | + | + | + | + | + | + | + | + | + | + | + | + | + | + | + | + | + | + | + | + | + | + |
| Mayo Checklist |  |  |  |  |  |  |  |  |  |  |  |  |  |  |  |  |  |  |  |  |  |  |  |  |  |  |  |
|  | 1. 1. Vowel or consonant errors including distorted substitutions. | + | + | + | + | + | + | + | + | + | + | + | + | + | + | + | + | + | + | + | + | + | + | + | + | + | + |
|  | 1. Intrusive *schwa*. | - | - | - | - | - | - | + | - | - | - | + | - | - | - | - | - | - | - | - | - | - | - | - | - | - | - |
|  | 1. Voicing errors. | + | + | + | + | - | - | + | - | + | - | + | + | + | + | + | + | + | + | - | - | + | - | + | + | + | + |
|  | 1. Lexical stress errors or equal stress. | + | - | - | + | - | - | + | - | - | + | + | - | + | + | - | - | + | - | - | + | + | - | - | + | + | - |
|  | 1. Syllable segregation. | + | + | + | + | + | + | + | + | - | + | + | + | + | + | - | + | + | + | + | - | + | + | - | + | + | + |
|  | 1. Slow rate | + | - | + | + | - | + | + | + | - | - | + | + | - | + | - | - | - | + | - | - | + | - | - | + | + | - |
|  | 1. Difficulty achieving initial articulatory configurations and transitions into vowels. | + | + | + | + | + | + | + | + | + | + | + | + | + | + | + | + | + | + | + | + | + | + | + | + | + | + |
|  | 1. Slow DDK rate. | + | + | + | + | + | - | + | + | + | + | + | + | + | + | + | + | + | + | + | - | + | + | - | + | + | - |
|  | 1. Groping (articulatory searching prior to phonation). | + | + | + | + | - | + | + | + | - | + | + | - | + | - | + | + | + | + | + | + | + | + | + | + | + | + |
|  | 1. Increased difficulty with longer or more phonetically complex words. | + | + | + | + | + | + | + | + | + | + | + | + | + | + | + | + | + | + | + | + | + | + | + | + | + | + |
