# Supplementary material for: Expanding the molecular landscape of childhood apraxia of speech: evidence from a single-center experience
Source: Front Neurosci. 2024 Sep 24;18:1396240. doi: 10.3389/fnins.2024.1396240 (PMC11459770; doi:10.3389/fnins.2024.1396240)

***Supplementary Material***

# Functional profiles of the four children with, *KAT6A*, *UBA6* and *ZFHX4* gene variants

*KAT6A*

Proband #27, a boy, tested positive for a *de novo* *KAT6A* variant. The child was born at term after an unremarkable pregnancy. At birth, his weight was 2980 g., length 48 cm., and cc 35 cm. As a newborn, he presented severe feeding difficulties that required medical attention but resolved within the first year. His parents described him as an infant with hypomimia, who showed very limited vocalization and babbling. He sat unsupported at 7 months and walked at 18 months. At the age of 3 years, he was first referred to speech therapy: at that time the child was non-verbal, although his social skills were normal. At 4 he underwent a first neurological examination, during which hypotonia and ligament hyperlaxity were observed. The child showed severe convergent strabismus that was corrected surgically; he had no heart condition and no epilepsy. His speech was severely disordered and characterized by the typical signs of CAS. Language assessment revealed moderate receptive impairment and more severe expressive morpho-syntactic and lexical difficulties. At the age of 8.5 years, when WES analysis was performed, the child had gained full intelligibility, although his speech continued to be characterized by dysprosody, scanning, devoicing and difficulties with longer words. Expressive grammar was found to be improved with the emergence of syntactically complex sentences, but difficulties in the use of free morphemes persisted. His expressive lexicon was severely impaired (z-score -5.54 on the BVL – Battery for Language Assessment for Children aged 4-12) (1), whilst receptive lexicon was only delayed (lexical quotient=78 on the Peabody Picture Vocabulary Test-Revised, Italian version) (2). Metaphonological skills (both global and phonemic) were < 5^th^ percentile (Assessment of Metaphonological Skills - CMF) (3). Academically, the child showed deficits in reading speed, accuracy, and comprehension, as well as persistent spelling errors (single word dictation <5^th^ percentile, Battery for the Assessment of Developmental Reading and Spelling Disorder) (4). He showed difficulties in mental calculation and in retrieving number facts. Handwriting was slow, effortful and imprecise. Cognitive profiling revealed a mild working memory deficit (WISC-IV, Working Memory Index=70).

*ZFHX4*

At the age of 13 years, proband #41, a boy, tested positive for a variation in *ZFHX4*, inherited from his father, who reported reading difficulties, but no speech and language disorder. The child was born at term after uneventful pregnancy. Early motor development was reported as normal, whilst early vocal skills were markedly delayed. The first words were produced after 4 years of age, despite normal social skills and intentionality. At the age of 7 his speech was still severely unintelligible. Expressive language was characterized by simple phrases with sporadic use of free morphemes, whilst receptive grammar and lexical skills were normal. Over time, expressive language improved, reaching normal levels at around 13 years, whilst speech remained slow, imprecise and dysprosodic. Academic skills were characterized by slow and inaccurate reading and spelling with graphomotor issues, but normal reading comprehension. Mathematical skills were age appropriate. Cognitive profiling revealed very good non-verbal fluid reasoning skills (WISC-IV, Perceptive Reasoning index=128), and working memory in the low normal range (WISC- IV, Working Memory Index=88).

*UBA6*

Probands #39 and #40 showed variations in the *UBA6* gene. Proband #39 inherited the variation from his father, who had a history of speech and language disorder not otherwise specified. Case #39 was born at term after unremarkable pregnancy and presented with normal early motor and social development. At birth his weight was 3420 g., length 49.5, and cc 34.5 cm. Gross motor landmarks were reached within the expected time, whilst speech acquisition was markedly delayed and slow. At age 6 his speech was still highly unintelligible, with a limited consonantal repertoire, dysprosodic, inaccurate and inconsistent on multisyllabic words. Expressive grammar was markedly altered: he produced complex sentences, but with omissions of free morphemes and difficulties with verb inflections. His receptive skills have always been age appropriate. At the time of the genetic diagnosis, 8 years of age, expressive language was characterized by residual errors in verbal inflections and in the use of free morphemes, and speech was still inaccurate, lacked fluency and was at times poorly intelligible. Reading skills were characterized by low accuracy, despite good speed and comprehension, and he produced numerous spelling errors. Cognitive profiling revealed weak working memory (WISC-IV, Working Memory Index=76). Proband #40, a boy, was born at term after an unremarkable pregnancy. At birth he weighed 2900 g. No conditions requiring medical attention were present after birth. He inherited a *UBA6* alteration from his mother, who did not report developmental speech and language delay, but was under medication for anxiety disorder. His older brother, who did not undergo genetic testing, showed speech and language impairment, as well as an academic learning disorder. In infancy, case #40 presented with normal feeding and gross motor skills, whilst babbling was scarce and poorly varied. The first simple words were produced by 18 months, and further development of speech was very slow, and production was poorly intelligible. At 3 years of age, he was able to produce a small set of approximated words. Lexical difficulties were still evident on further assessment at the age of 7.5 (Neuropsychological Lexical Assessment for Children, Cossu, 2013-1.2 SD) (5). By that age he had started to produce simple phrases and occasionally expanded sentences but had severe issues with the use of free and bound morphology. Receptive lexicon (PPVT-R lexical quotient=96) and receptive grammar (Grammar Comprehension Test for Children, +1.7 SD) (6) were normal. Speech was characterized by incomplete phonetic inventory, frequent errors, frequent inconsistencies, especially in multisyllabic words and connected speech, and incorrect stress assignment. Global motor skills, assessed using the Movement Assessment Battery for Children-2 (7), showed a mild overall delay, not consistent with developmental coordination disorder. Academically, mathematical abilities were normal and reading and spelling very delayed. Cognitive profiling revealed a mild working memory deficit (WISC-IV, Working Memory Index=67).

**Supplemental references**

1. Marini A, Marotta L, Bulgheroni S, Fabbro F, "Batteria per la Valutazione del Linguaggio in Bambini dai 4 ai 12 anni", Florence, Giunti OS, 2015.
2. Peabody Picture Vocabulary Test (PPVT-R - Dunn & Dunn, 1997); Italian version - Stella G, Pizzoli C, Tressoldi PE, Turin, Omega, 2000

Marotta L, Trasciani M, Vicari S, Test CMF - Valutazione delle Competenze Metafonologiche, Trento, Erickson, 2004

1. Sartori G, Job R, Tressoldi PE, Batteria per la Valutazione della Dislessia e della Disortografia Evolutiva - 2, Florence, Giunti Psychometrics, 2007
2. Cossu G, TNL Test Neuropsicologico Lessicale per l'Età Evolutiva, Florence, Hogrefe, 2013

Chilosi AM, Cipriani P, TCGB Test di Comprensione Grammaticale per Bambini. Tirrenia, Ed. Del Cerro, 1995

Henderson SE, Sugden DA, Barnett A, Movement Assessment Battery for Children, 2nd ed. London, Harcourt Assessment, 2007

**Supplementary Figure 1:** The operationalization of the assessment procedure to detect the speech features of CAS according to the Mayo 10-point checklist.

**
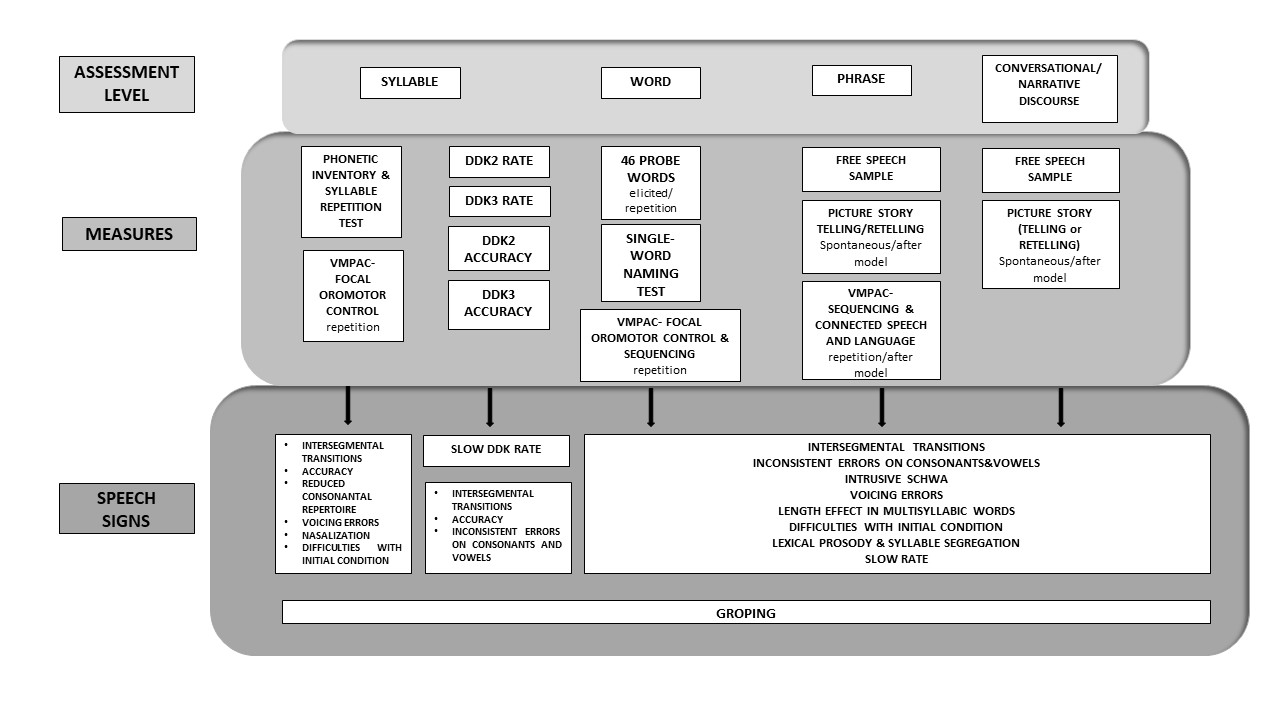
**

**Supplementary Figure 2:** Families analyzed by WES.

**Supplementary Figure 3: Research strategy to identify new CAS genes**. Germline variants were filtered step by step to pick up potentially interesting candidate CAS genes. First, the non-silent variants, including missense, nonsense, indels, and splice-site variants, were selected. Second, novel variants not already reported in GnomAD v2.1 were selected. Then, variants predicted to be pathogenic (Revel≥0.0644 and SpliceAI ≥0.2) were selected. After that, for five genes, the enrichment folds of the variants in CAS patients were calculated by means of comparison with the frequency in the control cohort, and Fisher’s exact test was performed to calculate the p-values. Finally, we identified potentially interesting genes based on significant p-values (≥0.005).


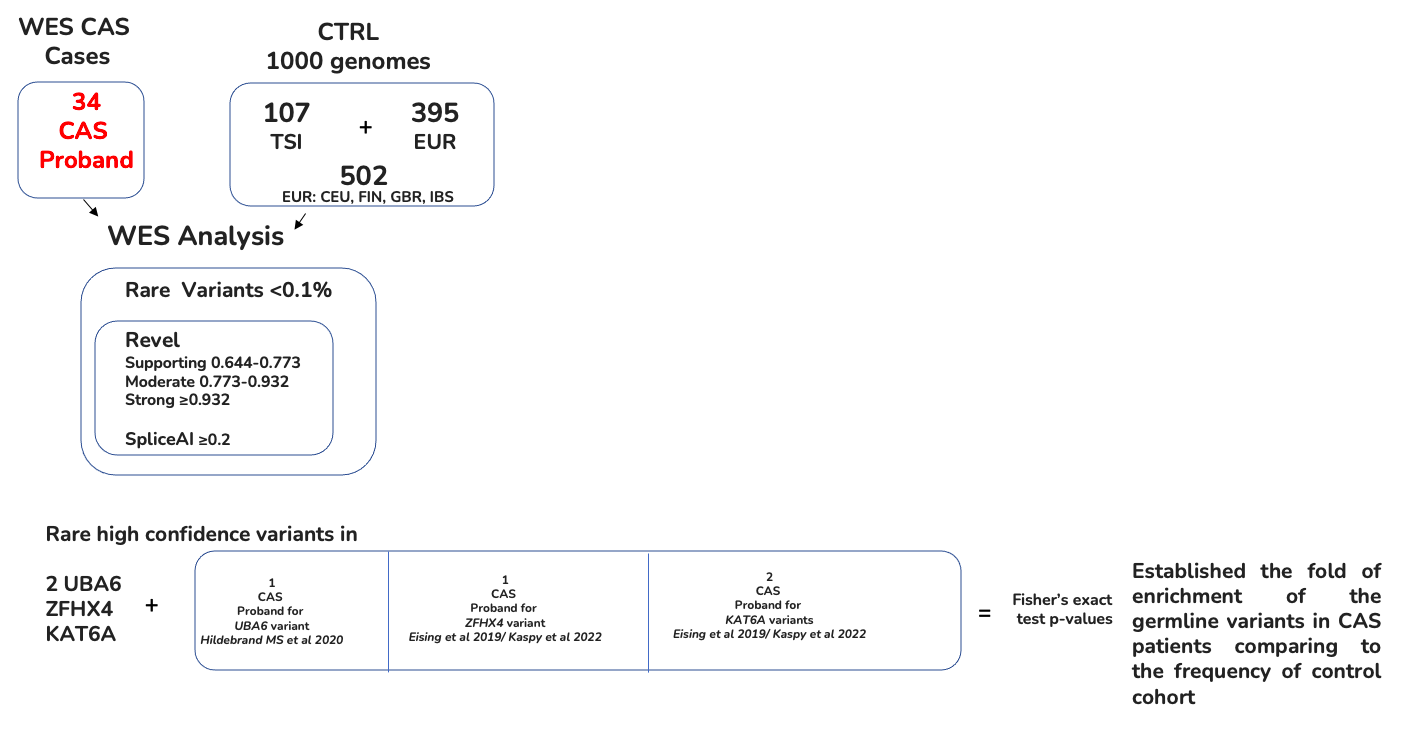


**Supplementary Figure 4:** RNA-seq RPKM values obtained from the BrainSpan database for the set of low-confidence (LC) genes. For each donor available in the BrainSpan Atlas of the Developing Human Brain1 database, the RNA-seq expression values of the LC genes are plotted using the Complex Heatmap 2 R package. The heatmap is divided into submodules, each representing the genes expression values for a given brain area, described at the top of the plot. Note that each donor is associated with one or more columns, since the RPKM values are available for multiple brain areas. Genes are also clustered based on their RPKM values, as represented by the dendrogram on the left, in order to better visualize expression similarity between genes**.**


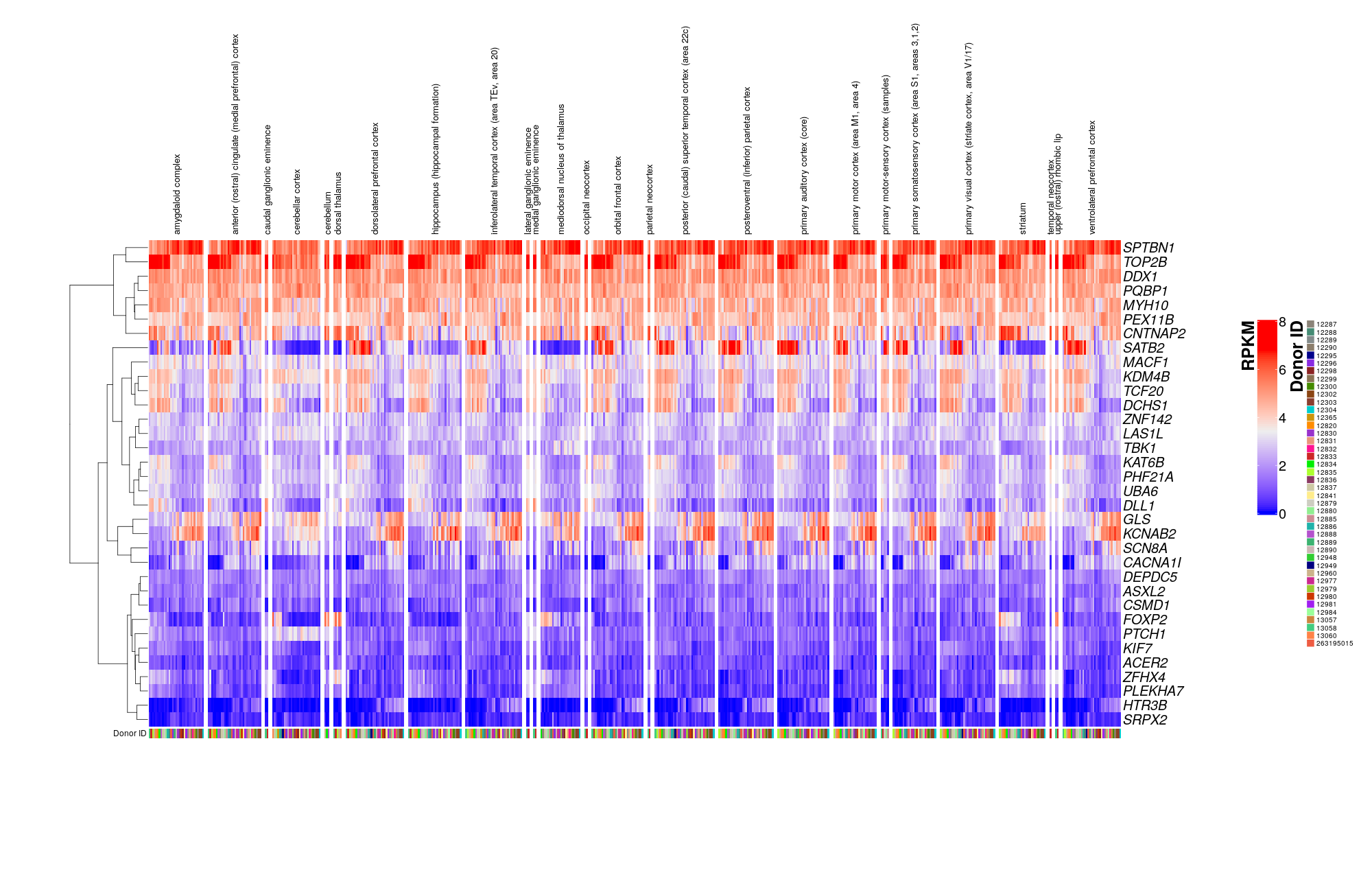

Supplement: Supplementary file 6 [file Data_Sheet_1.docx]
